# Supplementary material for: Diaphragm-based carbon monoxide electrolyzers for multicarbon production under alkaline conditions
Source: Nat Commun. 2025 Sep 26;16:8444. doi: 10.1038/s41467-025-63004-1 (PMC12475119; doi:10.1038/s41467-025-63004-1)
Supplement: Supplementary file 1 — Supplementary Information [file 41467_2025_63004_MOESM1_ESM.pdf]

Supplementary Information

Title:

Diaphragm-based Carbon Monoxide Electrolyzers for Multicarbon Production under Alkaline Conditions

Authors list:

Wanyu Deng[†,1], Siyang Xing[†,1], Guilherme Warwick Parker Maia[1], Zhaoxi Wang[1], Bradie S. Crandall[2], Feng Jiao[*,1]

Affiliations:

[1]Center for Carbon Management, Department of Energy, Environmental, and Chemical Engineering, McKelvey School of Engineering, Washington University in St. Louis, St. Louis, MO, USA

[2]Lectrolyst, Wilmington, DE, USA

†These authors contribute equally to this work.

*Email: jiaof@wustl.edu

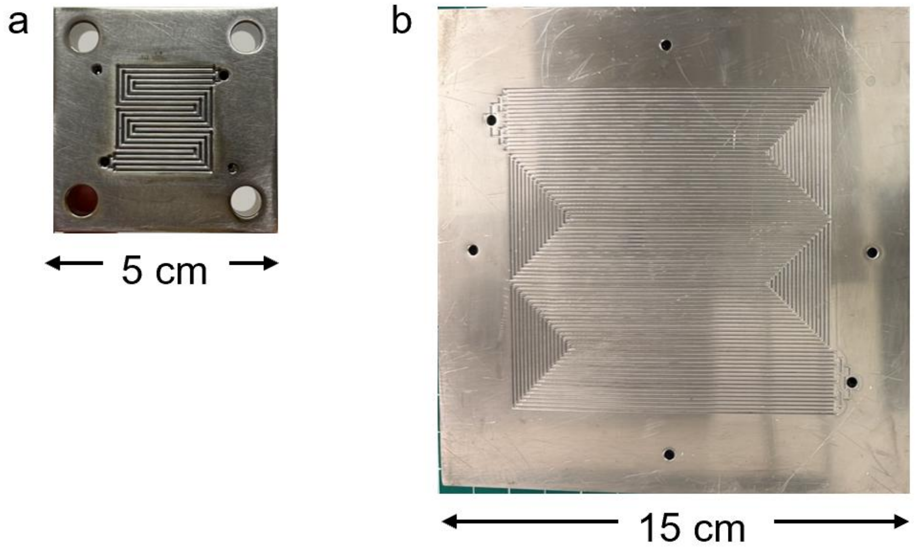

Supplementary Fig. 1 | Photographs of (a) 5 cm^2 and (b) 100 cm^2 stainless steel endplates.

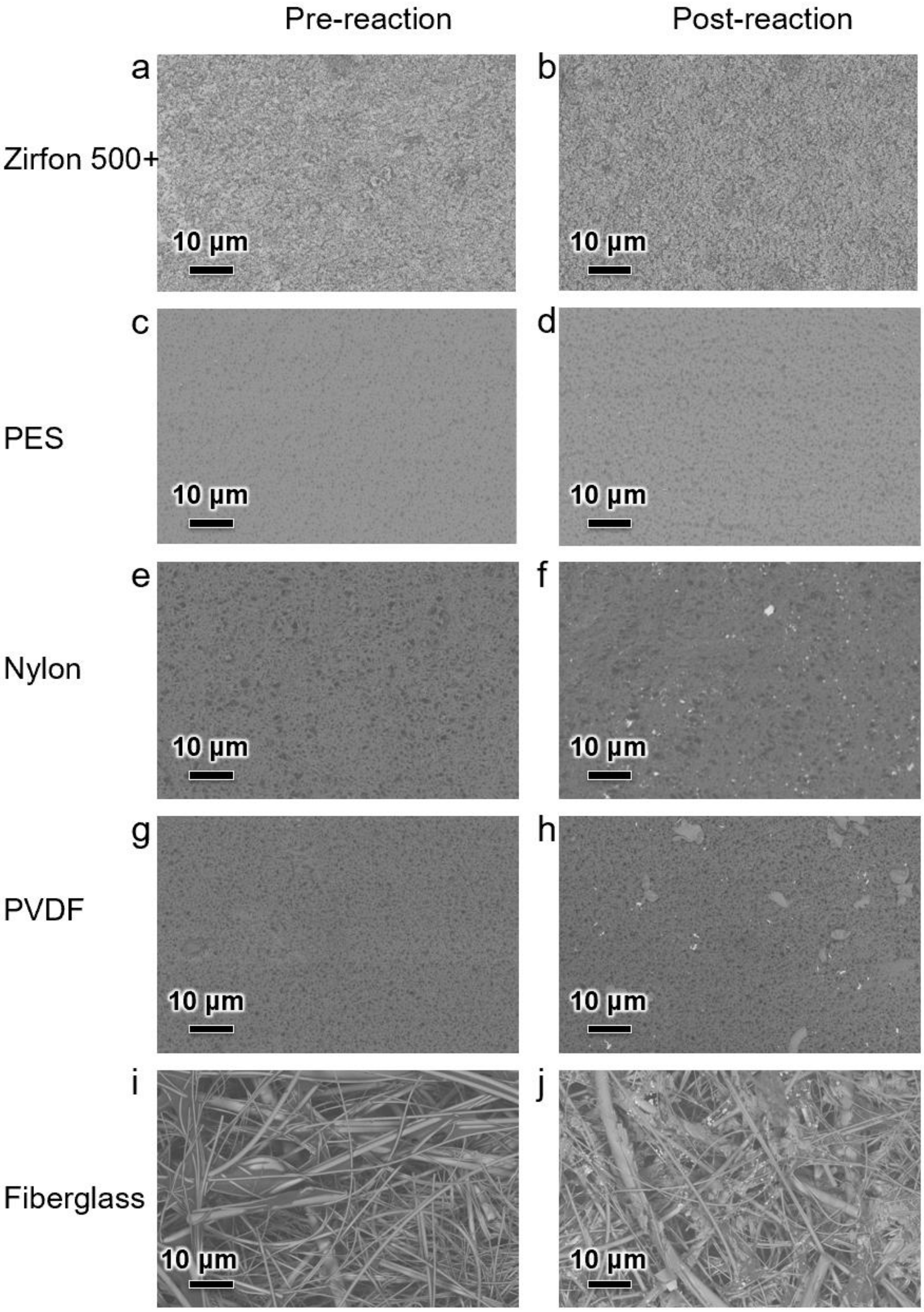

Supplementary Fig. 2 | Scanning electron microscopy (SEM) images of (a, b) Zirfon 500+, (c, d) Polyethersulfone (PES), (e, f) Nylon, (g, h) Polyvinylidene fluoride (PVDF), (i, j) Fiberglass diaphragm before and after 120 h test. The cell was operated at a fixed current density of 200 mA cm^{-2} using a 1 M KOH electrolyte at 3 mL min^{-1}, a 40–60 nm Cu nanoparticle cathode, a $NiFeO_x$/Ni foam anode, with CO fed at a rate of 30 sccm. Due to the short circuit caused by structural damage, Fiberglass was only tested for 24 hours.

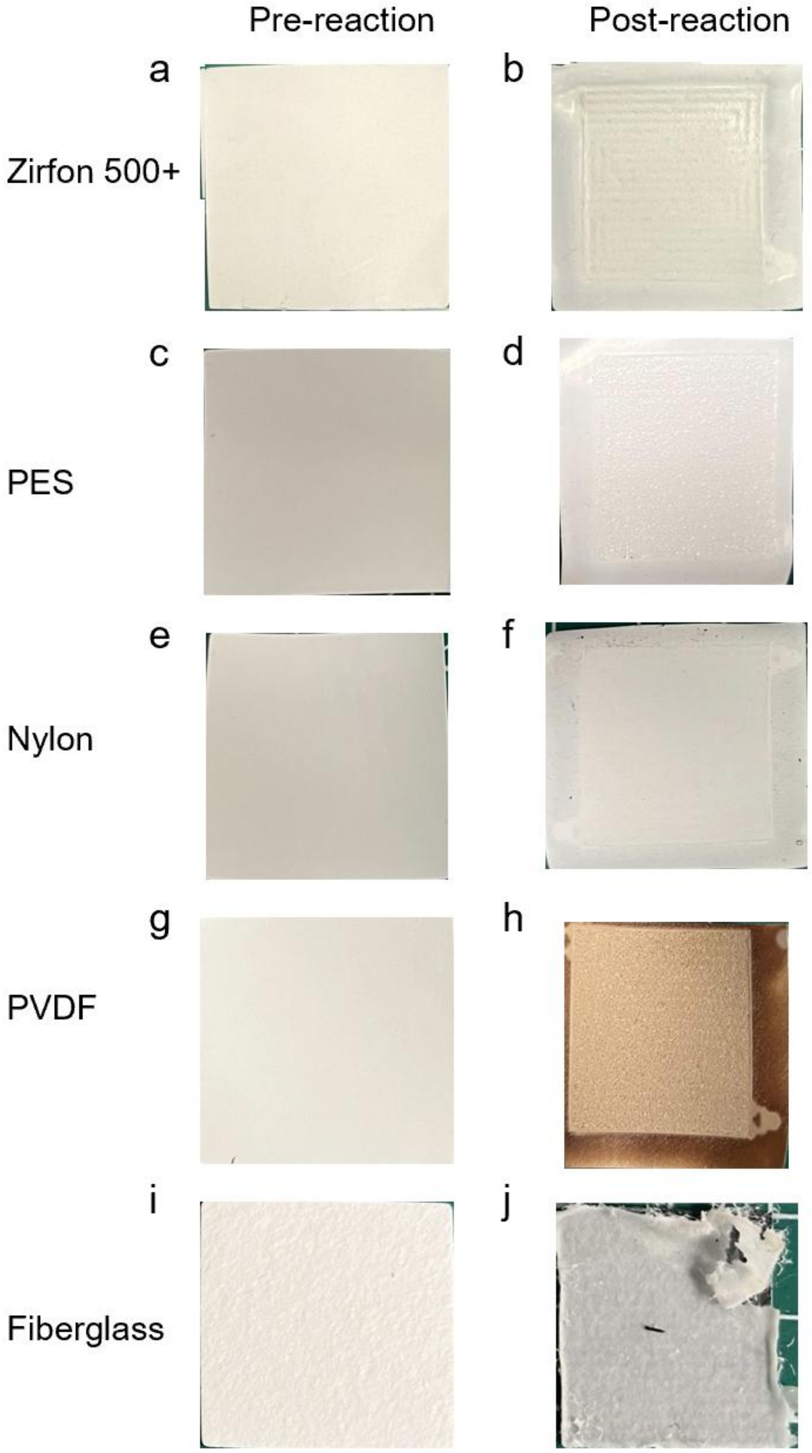

Supplementary Fig. 3 | Photograph of Zirfon, PES, Nylon, PVDF, and Fiberglass diaphragm before and after 120 h test. The cell was operated at a fixed current density of 200 mA cm^{-2} using a 1 M KOH electrolyte at 3 mL min^{-1}, a 40–60 nm Cu nanoparticle cathode, a $NiFeO_x$/Ni foam anode, with CO fed at a rate of 30 sccm. Due to the short circuit caused by structural damage, Fiberglass was only tested for 24 hours.

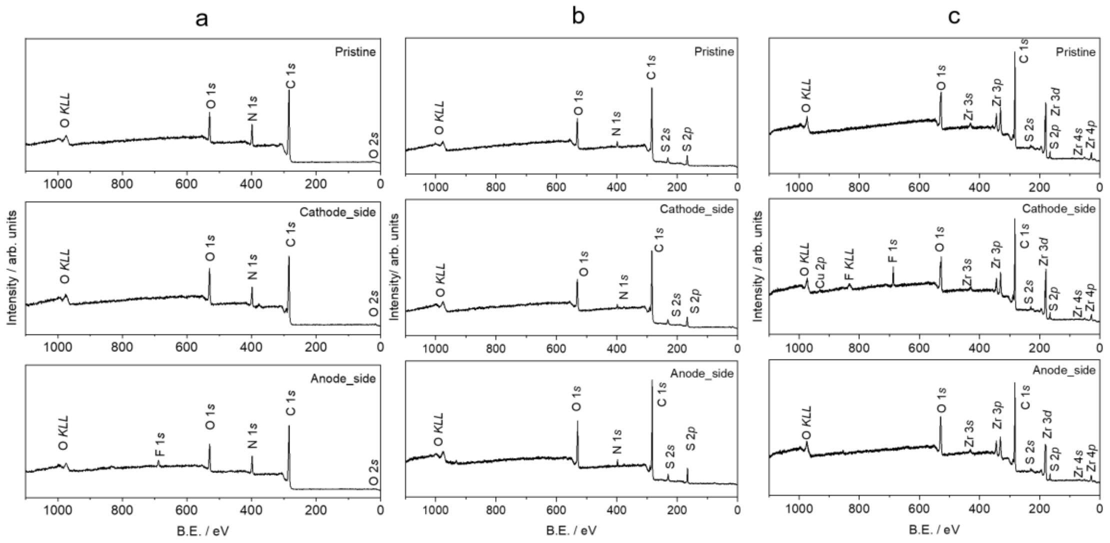

Supplementary Fig. 4 | X-ray photoelectron spectroscopy (XPS) survey spectra comparison of (a) Nylon, (b) PES, and (c) Zirfon 500+ before and after 120 h test: Pristine samples, Cathode-side, and Anode-side of post-reaction diaphragms. The cell was operated at a fixed current density of 200 mA cm^{-2} using a 1 M KOH electrolyte at 3 mL min^{-1}, a 40–60 nm Cu nanoparticle cathode, a $NiFeO_x$/Ni foam anode, with CO fed at a rate of 30 sccm.

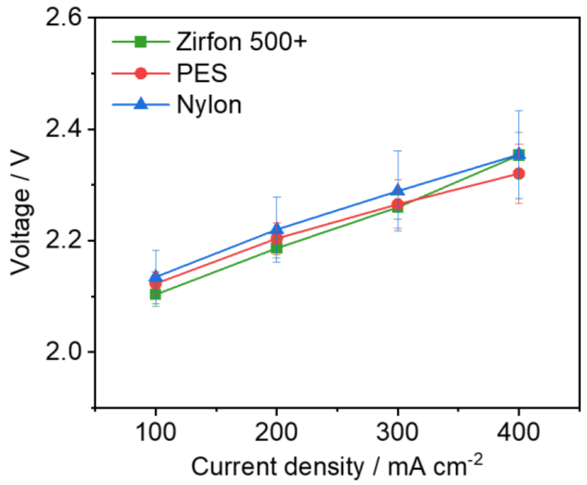

Supplementary Fig. 5 | Voltage comparison of CO electroreduction (COR) performance with different diaphragms among different current density. The data presented here are from the same experiments shown in Fig. 2b of the main text. Error bars represent the standard deviation from three independent measurements.

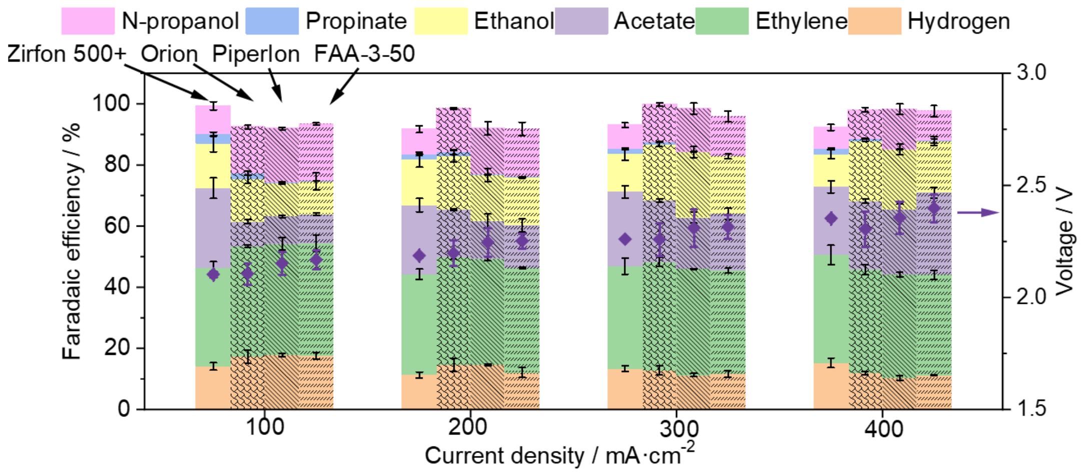

Supplementary Fig. 6 | Faradaic efficiency for all detectable products and corresponding cell voltages measured using Zirfon 500+ and anion-exchange membranes (AEMs) among different current densities. The 5 cm^2-cell was operated at different current densities using a 1 M KOH electrolyte at 3 mL min^{-1}, a 40–60 nm Cu nanoparticle cathode, a $NiFeO_x$/Ni foam anode, with CO fed at a rate of 30 sccm. Error bars represent the standard deviation from three independent measurements.

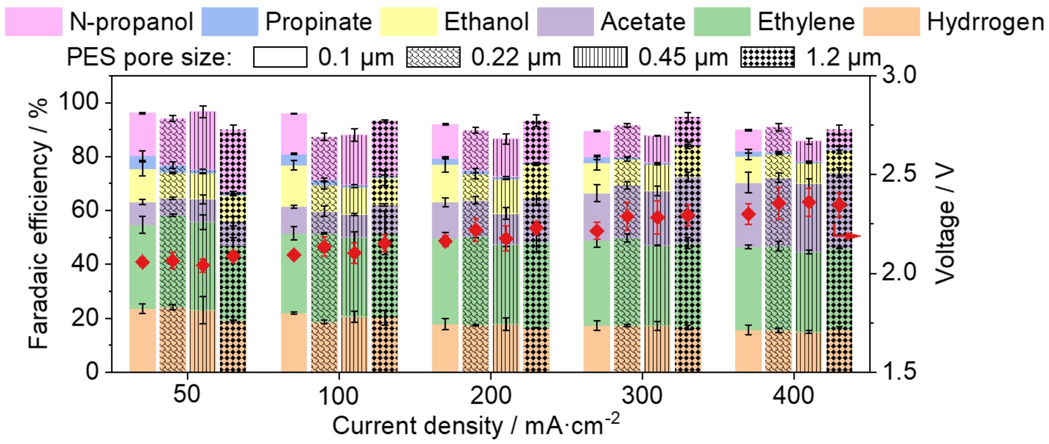

Supplementary Fig. 7 | Faradaic efficiency for all detectable products and corresponding cell voltages measured using different pore size PES diaphragms among different current densities. When the pore size increased to 5 μm, the cell experienced a short circuit, and no CO product can be detected. The 5 cm^2-cell was operated at different current densities using a 1 M KOH electrolyte at 3 mL min^{-1}, a 40–60 nm Cu nanoparticle cathode, a $NiFeO_x$/Ni foam anode, with CO fed at a rate of 30 sccm. Error bars represent the standard deviation from three independent measurements.

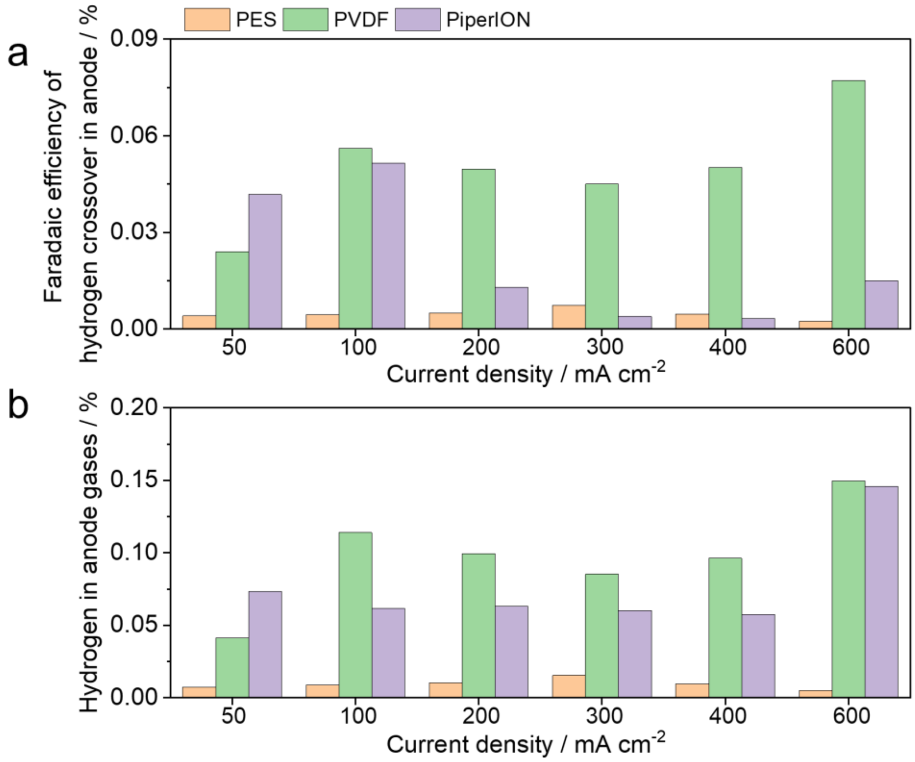

Supplementary Fig. 8 | Comparison of hydrogen crossover from the cathode to the anode (a) Faradaic efficiency and (b) volume percentage for the membrane and diaphragms under varying current densities. **No hydrogen was detected in Zirfon 500+ based cell.** The 5 cm^2-cell was operated at different current densities using a 1 M KOH electrolyte at 3 mL min^{-1}, a 40–60 nm Cu nanoparticle cathode, a $NiFeO_x$/Ni foam anode, with CO fed at a rate of 30 sccm, back pressure 16.5 psi.

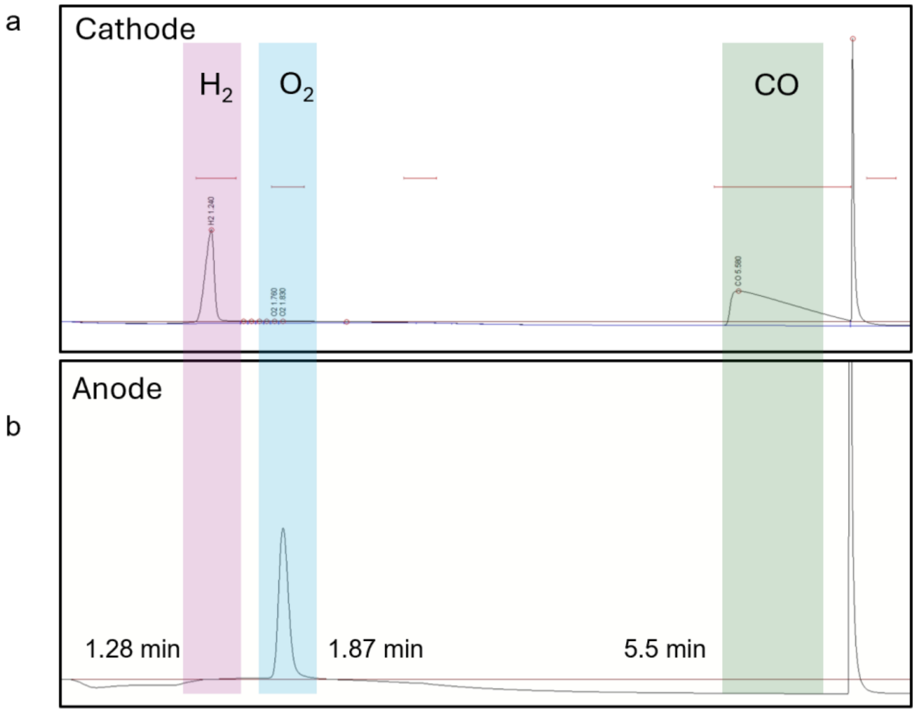

Supplementary Fig. 9 | GC results of (a) cathode and (b) anode gas products from Zirfon 500+ electrolyzer at 600 mA cm^{-2}. No hydrogen was detected at the anode and no oxygen at the cathode, indicating the absence of detectable gas crossover in the system. The 5 cm^2-cell was operated at various current densities using 1 M KOH (3 mL min^{-1}), with a 40–60 nm Cu nanoparticle cathode and a $NiFeO_x$/Ni foam anode. CO was supplied to the cathode at 30 sccm under a back pressure of 16.5 psi. Anode gas products were carried out for analysis by flowing 30 sccm Ar to purge the anode chamber into the GC.

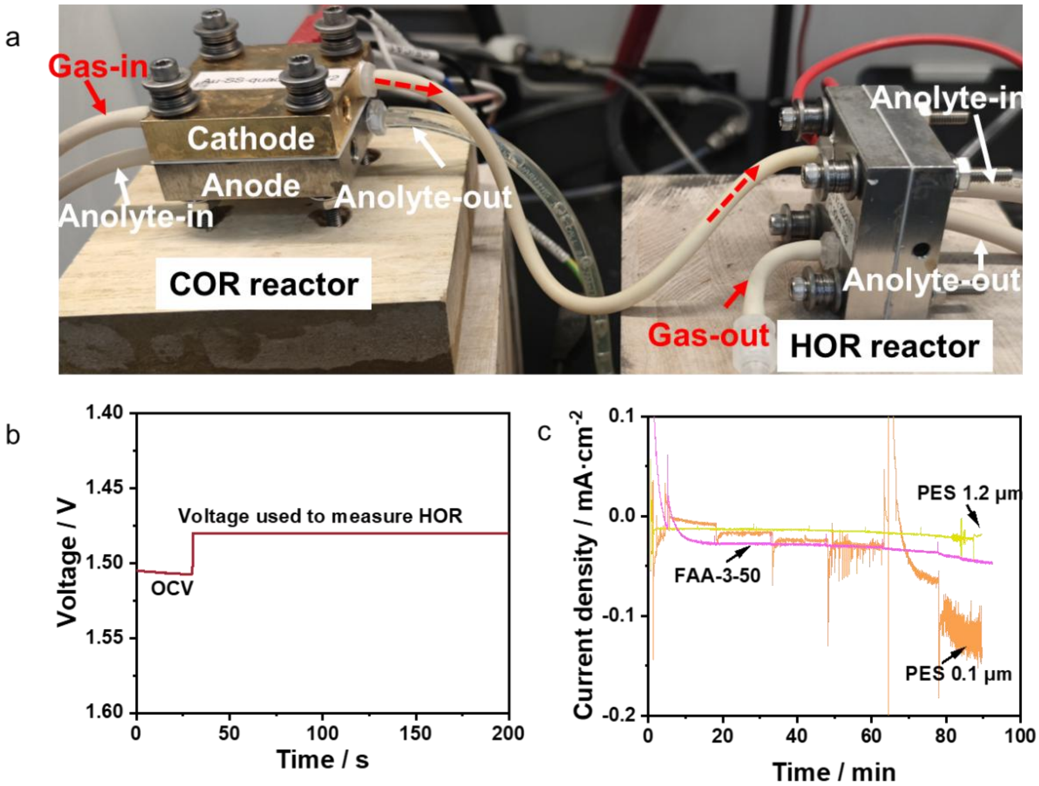

Supplementary Fig. 10 | (a) Photographs of the COR and hydrogen oxidation reaction (HOR) reactors used for crossover analysis. (b) Applied voltage profile of 1.48 V during HOR testing, which is slightly below the open-circuit potential (~1.52 V). (c) Corresponding current response of the HOR reactor. The relatively low current indicates minimal hydrogen crossover. For the COR electrolyzer, the 5 cm^2-cell was operated at 400 mA cm^{-2} using 1 M KOH (3 mL min^{-1}), with a 40–60 nm Cu nanoparticle cathode and a $NiFeO_x$/Ni foam anode. CO was supplied to the cathode at 30 sccm under a back pressure of 16.5 psi. For the HOR reactor, the 5 cm^2-cell was operated at 1.48V using 1 M KOH (3 mL min^{-1}), with a 40–60 nm Cu nanoparticle cathode and a $NiFeO_x$/Ni foam anode. The outlet gases from COR electrolyzer was supplied to the cathode under a back pressure of 16.5 psi.

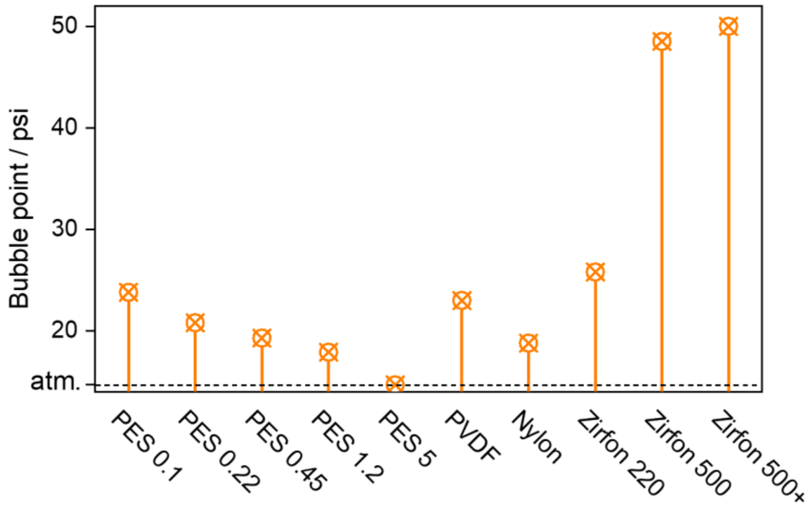

Supplementary Fig. 11 | Bubble point comparison of different diaphragms. Dash line shown in this figure represents atmospheric pressure. More experiment details could be found at Method gas crossover experiments section.

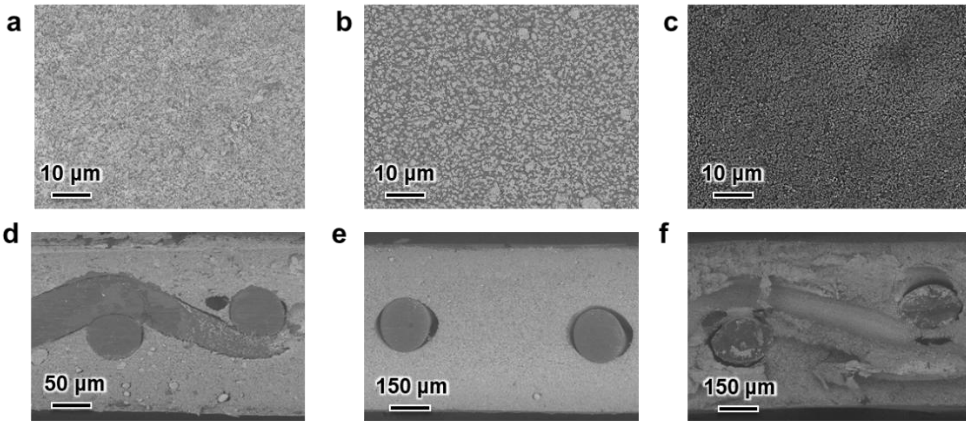

Supplementary Fig. 12 | SEM images of Zirfon diaphragms from surface:(a) Zirfon 220, (b)Zirfon 500, (c)Zirfon 500+ and cross section:(d) Zirfon 220, (e)Zirfon 500, (f)Zirfon 500+. Those SEM images is consistent with the information from the Agfavendor.

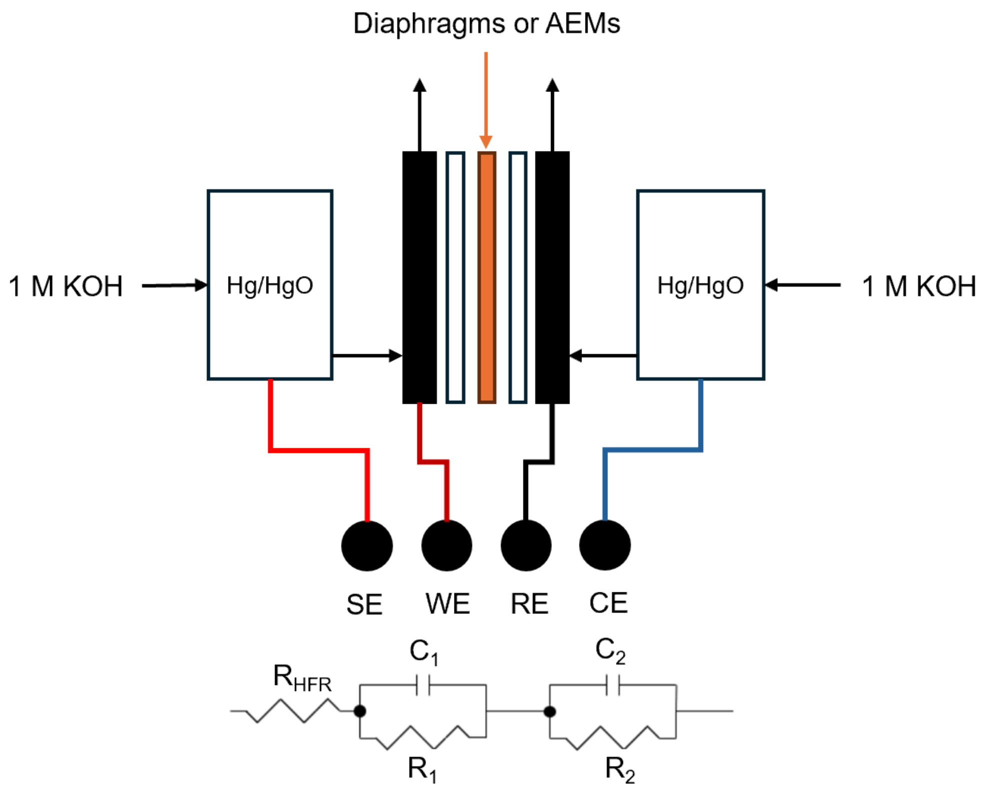

Supplementary Fig. 13 | Schematic of four electrode set-up for membrane impedance testing. Equivalent circuit is provided. WE: Working electrode. SE: Sensing electrode. RE: Reference electrode. CE: Counter electrode

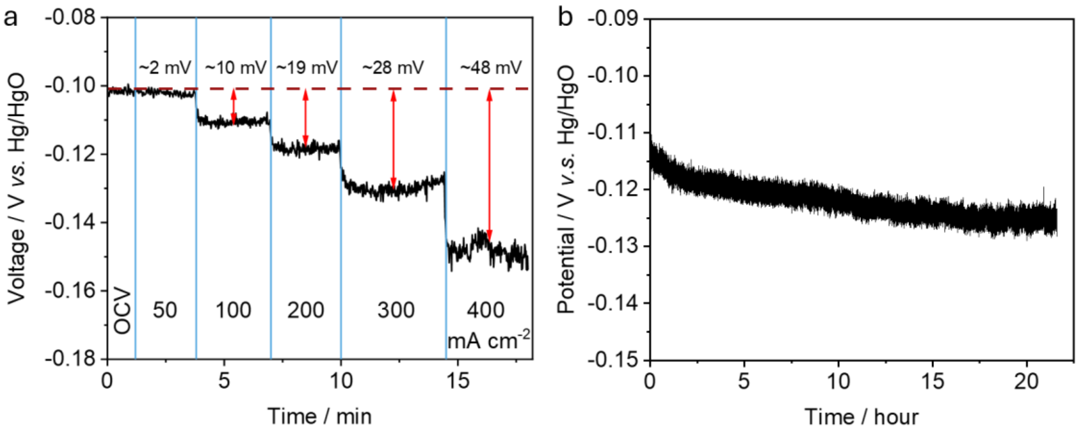

Supplementary Fig. 14 | a. Anode nickel foam quasi-reference electrode potential at varying current densities. b. Stability assessment of the nickel foam quasi-reference electrode at 200 mA cm^{-2}.

To evaluate the stability of nickel foam as a quasi-reference electrode in the CO electrolyzer, electrode potential measurements were conducted under varying current densities as well as under prolonged operation at a constant current density of 200 mA cm^{-2}. For the current density-dependent test, potentials of anode nickel foam quasi-reference were recorded at 50–400 mA cm^{-2}, and the observed fluctuation in electrode potential was consistently within 50 mV *vs.* Hg/HgO. These results indicate that the integrated nickel foam quasi-reference electrode provides sufficient accuracy for potential measurements in the context of the electrochemical cell.[1]

Additionally, the potential stability of the nickel foam reference electrode was evaluated over 20 hours at 200 mA cm^{-2}. The measured potential variation was less than 20 mV *vs.* Hg/HgO during the entire test period, further confirming that nickel foam can serve as a reliable quasi-reference electrode for CO electrolysis applications.

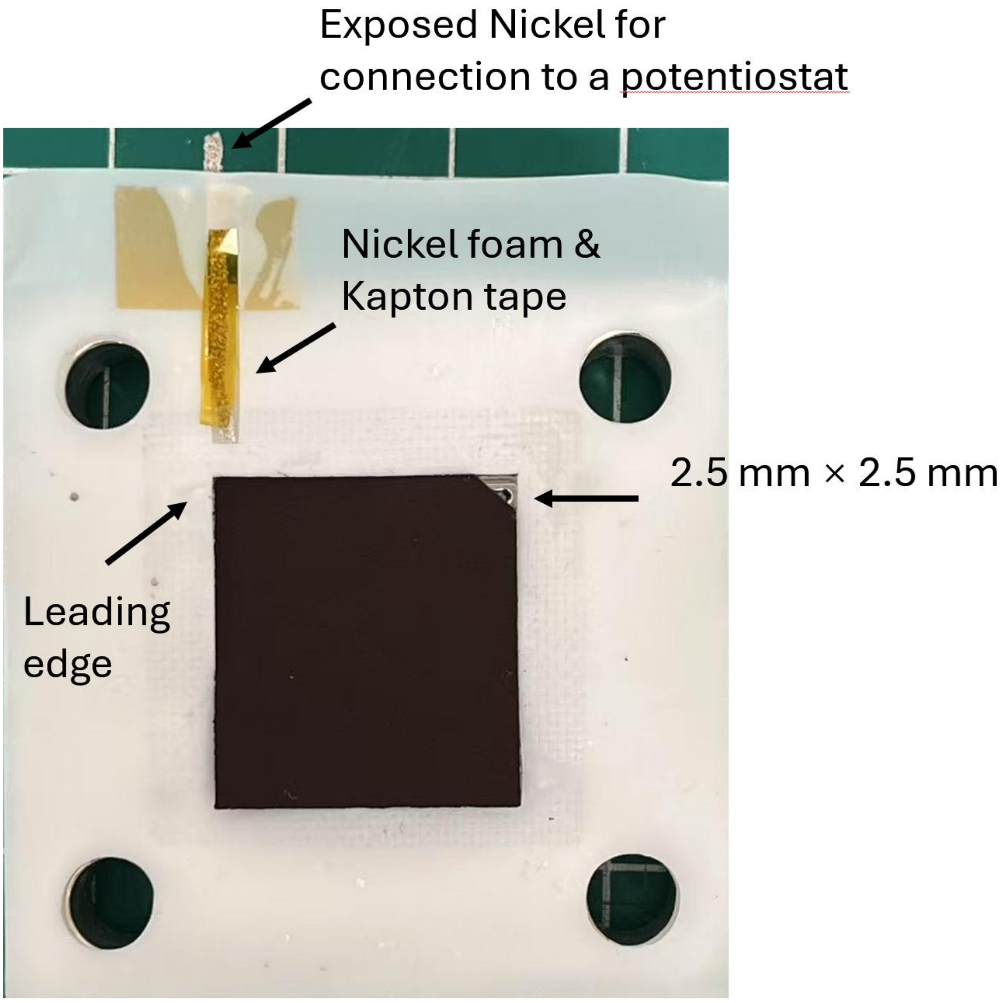

Supplementary Fig. 15 | Photograph of quasi-reference electrode positioning in the electrochemical cell and corner-cut catalyst.[2]

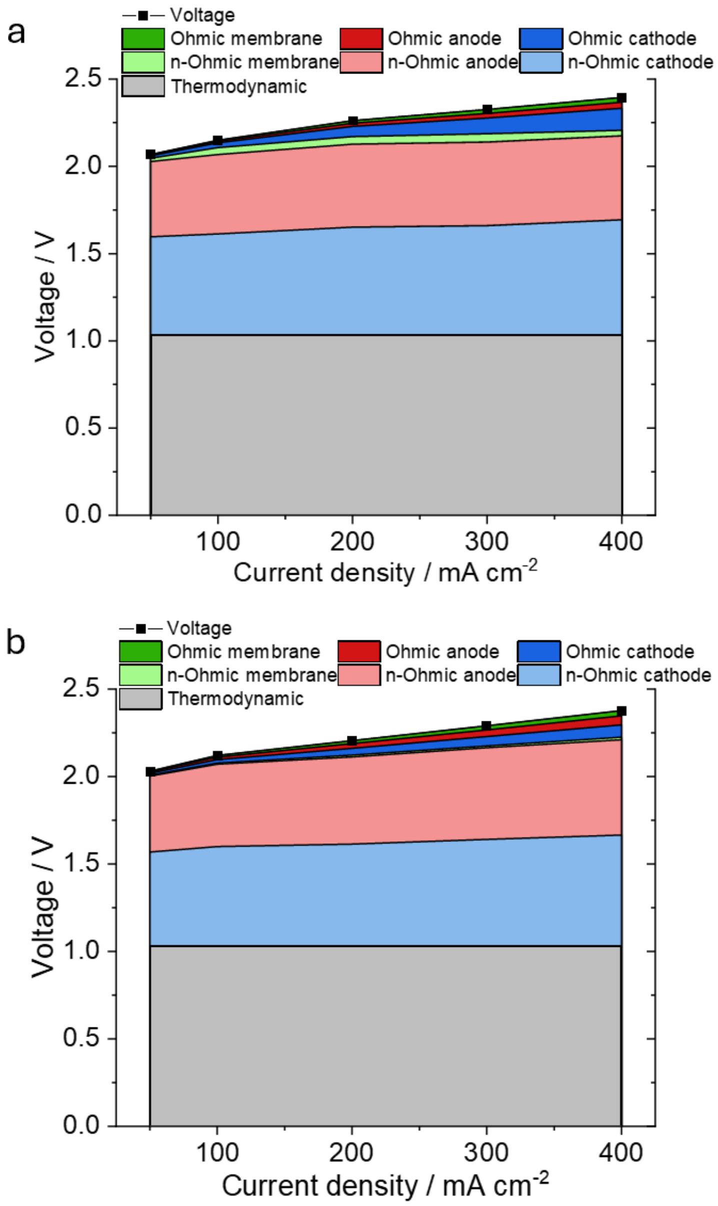

Supplementary Fig. 16 | Voltammetric analysis of COR performance, with breakdown of ohmic and non-ohmic overpotentials for (a) PiperION and (b) Zirfon 500+ systems.

Under galvanostatic control, the potentials of the cathode ($E_{cathode}$), anode (E_{anode}), and membrane ($E_{membrane}$) can be measured by measuring the voltage drop between current collectors and reference electrodes pressed on the membrane (eq 1). When the potential of the reference electrode under operating conditions is known, contributions from the expected thermodynamic potential, E_{th}, and inefficiencies manifested as overpotentials, η_i, can be backed out (eq 2). Primary sources of overpotential in this system are related to ion/electron transport (η_{cond}), electrode kinetics (η_{kin}), and mass-transport-induced thermodynamic/kinetic effects (η_{mt}) (eq 3). Typically, ion/charge conduction overpotential both in the bulk and in the thin-film electrolyte, generally established to

be ohmic (R_{cond}), can be extracted with relative ease by measuring impedance at a fast frequency (>10^3 Hz).[3] The combined kinetic/mass-transport overpotential ($\eta_{non-ohmic}$) at the operating current density, i'', can be evaluated by subtracting the ohmic resistance (eq 4). For generality, resistances are normalized to the active area of the electrode.

$$E_{cell} = E_{cathode} - E_{anode} + E_{membrane} \quad (1)$$

$$E_i = E_{i,th} + \eta_i \quad (2)$$

$$\eta_i = \eta_{i,kin} + \eta_{i,mt} + \eta_{i,cond} \quad (3)$$

$$\eta_{non-ohmic} = \eta_{i,kin} + \eta_{i,mt} = \eta_i - i''R_{cond} \quad (4)$$

Based on the above analysis, the overall cell voltage was deconvoluted into seven components using a five-electrode configuration. These include ohmic overpotentials associated with ion/electron transport in the cathode, anode, and membrane regions (denoted as ohmic cathode, ohmic anode, and ohmic membrane, respectively), as well as non-ohmic overpotentials arising from reaction kinetics and mass-transport-induced thermodynamic or kinetic limitations (denoted as n-ohmic cathode, n-ohmic anode, and n-ohmic membrane). In addition, the required thermodynamic potential is included as a baseline for comparison.

The polarization curve shown in this figure was directly measured by the potentiostat. When compared with the sum of electrode potentials obtained from the five-electrode deconvolution, an excellent match was observed between the two methods. A detailed breakdown of each overpotential contribution is provided in Supplementary Fig. 17.

The Zirfon-based cell exhibited a lower cathodic non-ohmic potential (Supplementary Fig. 17), which can be attributed to the higher compressibility of the Zirfon diaphragm (Supplementary Fig. 18), allowing better interfacial contact with the cathode and thereby reducing contact resistance.[2] In contrast, the anodic non-ohmic potential was slightly higher in the Zirfon-based cell (Supplementary Fig. 17), possibly due to its larger water contact angle (Supplementary Fid. 19), which may promote gas bubble accumulation at the diaphragm–anode interface and increase interfacial resistance. In summary, a higher area-specific resistance of Zirfon does not significantly impact the overall cell voltage. Instead, the interfacial contact differences introduced by its physical properties in the MEA system appear to play a more prominent role.

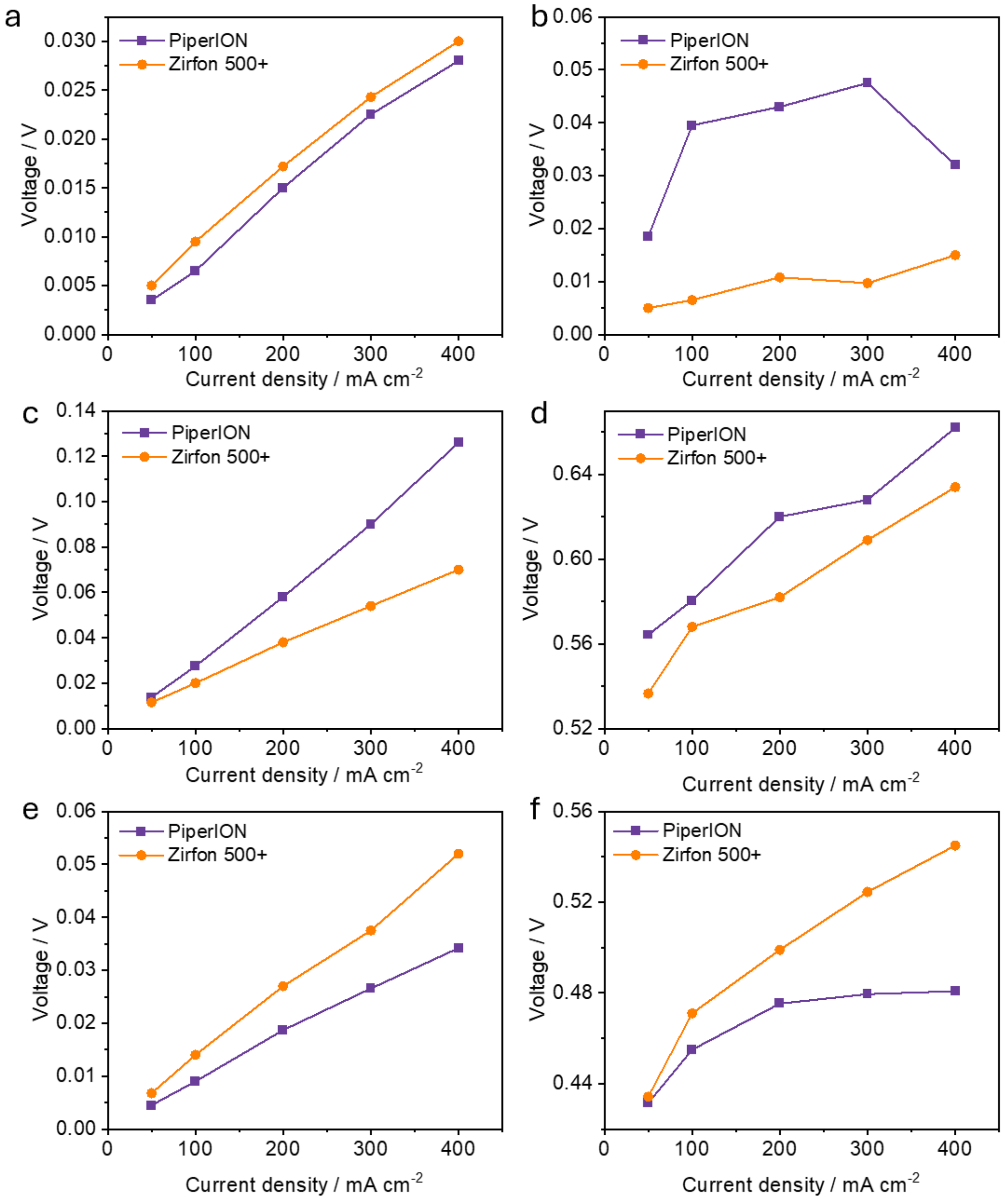

Supplementary Fig. 17 | Breakdown of the cell voltage into ohmic and non-ohmic overpotentials for the membrane, cathode, and anode components in the COR system, as a function of current density. The data were obtained via five-electrode measurements: (a) ohmic overpotential cross membrane/diaphragm, (b)non-ohmic overpotential cross membrane/diaphragm, (c) ohmic overpotential from cathode, (d) non-ohmic overpotential from cathode, (e) ohmic overpotential from anode, and (f) non-ohmic overpotential from anode. See method section for the experimental detail.

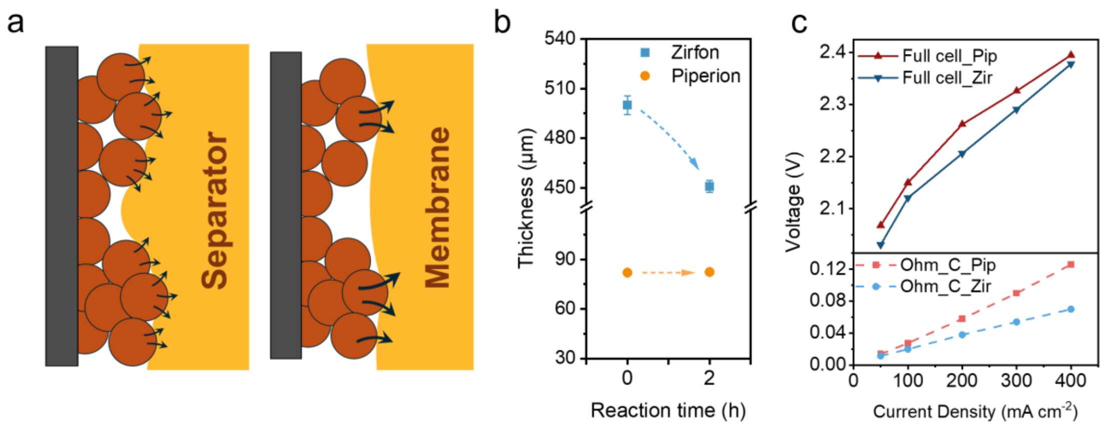

Supplementary Fig. 18 | Comparison of catalyst-membrane/separator interface. **a** | Scheme of catalyst-membrane/separator interface contact condition. **b** | Thickness measurement of separator and membrane before compression and after reaction. **c** | Cathode ohmic overpotential and corresponding cell voltages for different current density.

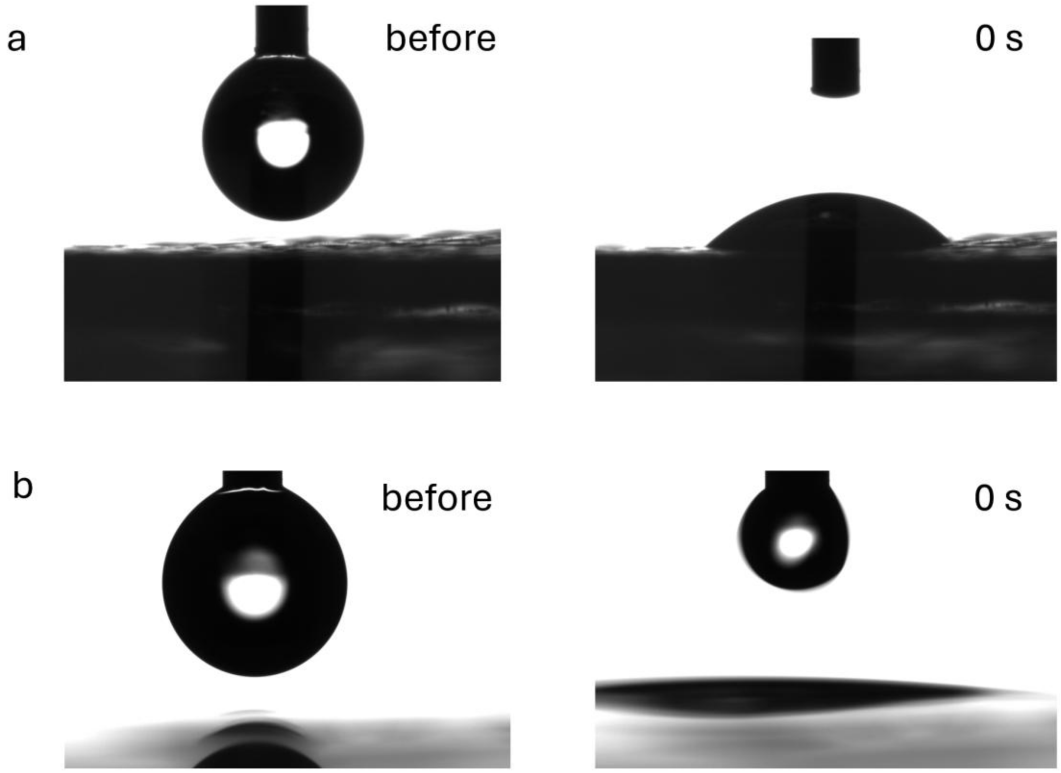

Supplementary Fig. 19 | Water contact angle measurements on (a) fresh Zirfon 500+ and (b) activated PiperION membranes before and immediately upon water droplet contact.

When a water droplet was placed on fresh Zirfon 500+, it exhibited a contact angle of approximately 41°, whereas on the activated PiperION membrane, the droplet was instantly and completely absorbed, preventing any measurable contact angle. This indicates that the activated PiperION possesses significantly superior hydrophilicity compared to Zirfon 500+.

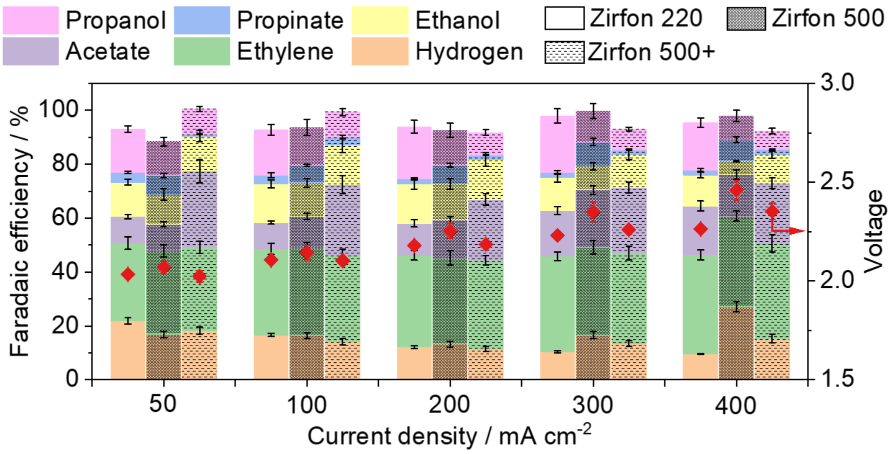

Supplementary Fig. 20 | Faradaic efficiency for all detectable products and corresponding cell voltages measured using different Zirfon diaphragms among different current densities. For the COR electrolyzer, the 5 cm^2-cell was operated at 400 mA cm^{-2} using 1 M KOH (3 mL min^{-1}), with a 40–60 nm Cu nanoparticle cathode and a $NiFeO_x$/Ni foam anode. CO was supplied to the cathode at 30 sccm under a back pressure of 16.5 psi. Error bars represent the standard deviation from three independent measurements.

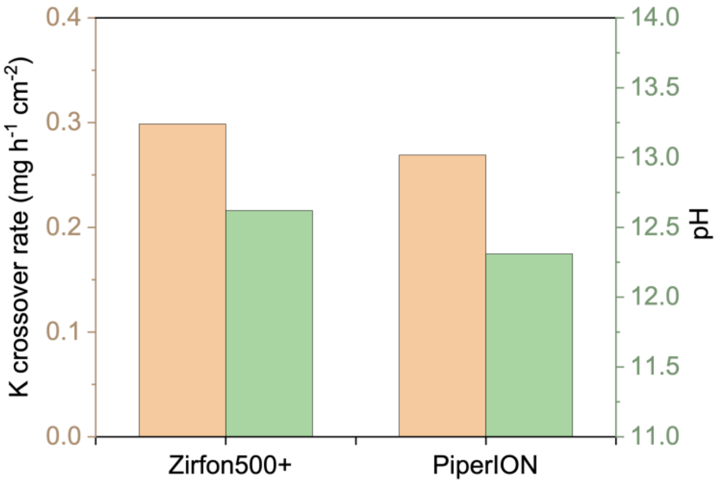

Supplementary Fig. 21 | Comparison of K^+ crossover rate and cathodic trap pH between Zirfon- and PiperION-based cells. To investigate differences in cathodic product purity between the Zirfon-based and PiperION-based cells, we performed 24-hour electrolysis experiments at a current density of 200 mA cm^{-2} in both systems. The downstream trap solution on the cathode side was collected and analyzed to assess the purity of the products. Initially, the trap contained 3 mL of Milli-Q water with a pH of 6.9. After 24 hours of operation, the pH of the trap increased to 12.62 for the Zirfon-based cell and to 12.31 for the PiperION-based cell. Under the influence of the electric field and concentration gradients, K^+ ions migrate toward the cathode and are subsequently carried into the trap solution by the gas stream, thereby affecting product purity. We therefore quantified the K^+ crossover rates in both systems. The K^+ crossover rate in the Zirfon-based cell (0.299 mg h^{-1} cm^{-2}) was found to be slightly higher than that in the PiperION-based cell (0.269 mg h^{-1} cm^{-2}), which can be attributed to the porous nature of the diaphragm in contrast to the denser structure of the AEM. Nevertheless, the difference between the two was relatively small, further confirming that Zirfon offers sufficiently low anolyte crossover and can effectively maintain compartmental separation in alkaline CO electrolysis.

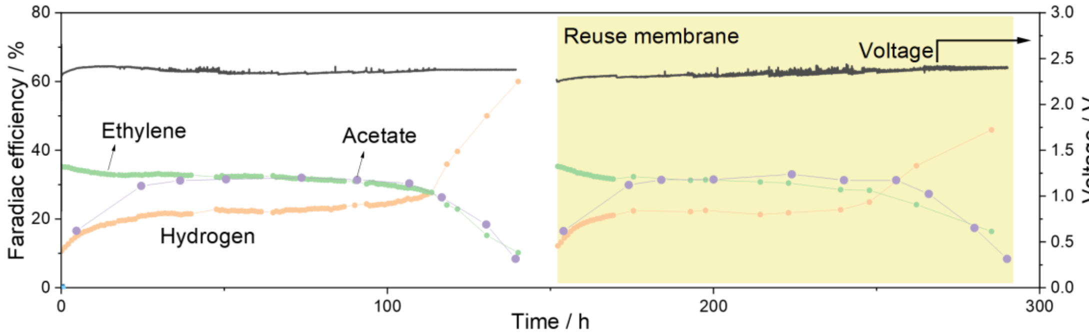

Supplementary Fig. 22 | Faradaic efficiency of acetate, ethylene and hydrogen and corresponding cell voltages measured using PiperION at a fixed current density of 200 mA cm^{-2}. The 5 cm^2 zero-gap CO electrolyzer was operated at room temperature with a 1 M KOH electrolyte at 3 mL min^{-1}, a 40–60 nm Cu nanoparticle cathode, a $NiFeO_x$/Ni foam anode, with CO fed at a rate of 50 sccm.

The sharp increase in hydrogen evolution in the later stage is not likely related to the membrane degradation. This is supported by the fact that reused membranes could still deliver comparable performance to pristine ones in subsequent. Specifically, we suspect this is caused by the interfacial issues between the cathode and the PiperION membrane (Supplementary Fig. 18). The higher interfacial resistance in this system may lead to uneven electric field distribution across the electrode surface, accelerating localized deactivation.

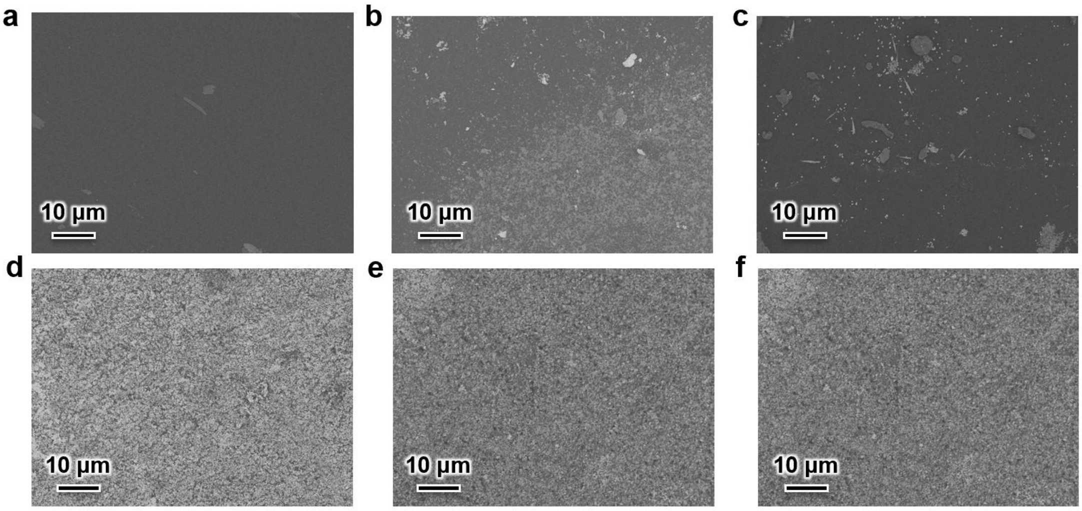

Supplementary Fig. 23 | SEM of pre-reaction PiperION and Zirfon (a, d) and of post-reaction PiperION and Zirfon towards anode (b, e) and cathode (c, f) side.

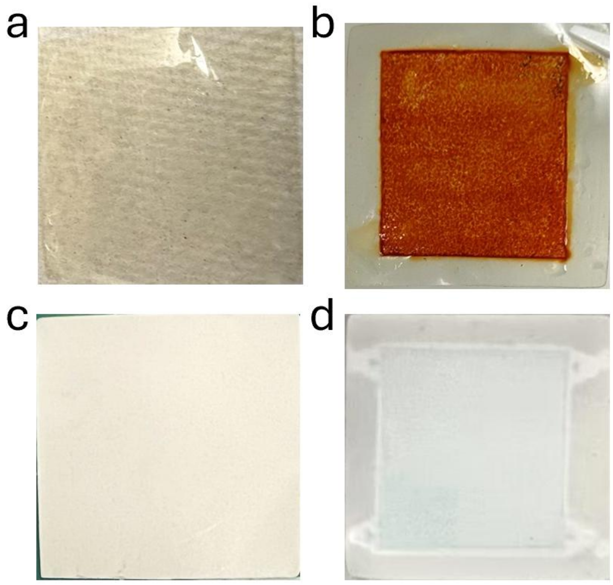

Supplementary Fig. 24 | Photographs of PiperION (a, b) and Zirfon 500+ (c, d) from pre- and post-reaction, respectively.

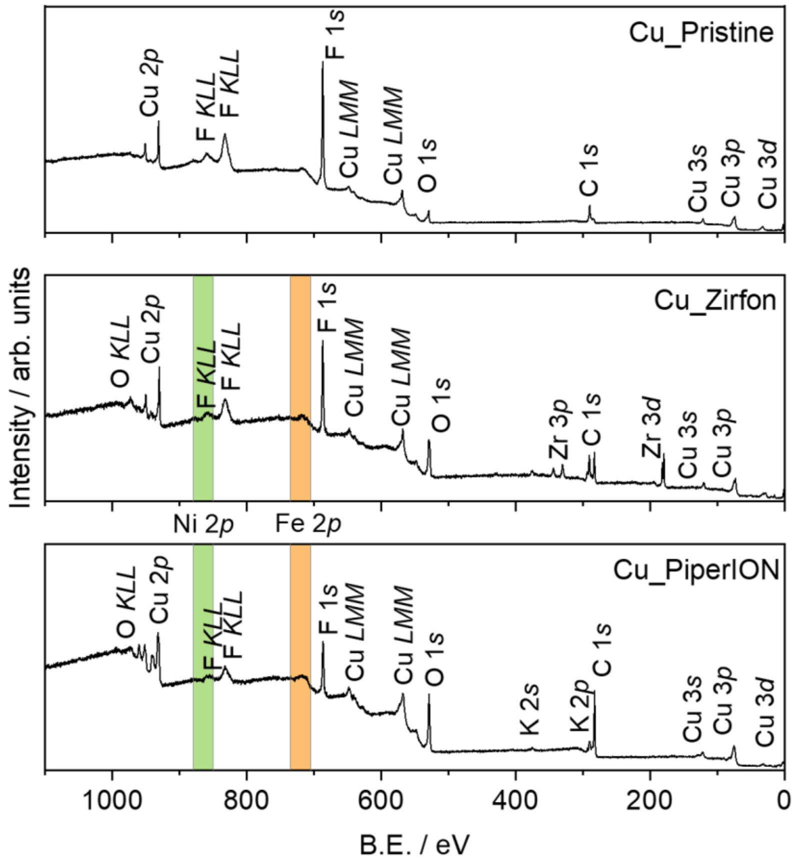

Supplementary Fig. 25 | XPS survey spectra of the cathode before and after (from Zirfon 500+ and PiperION, respectively) stability test.

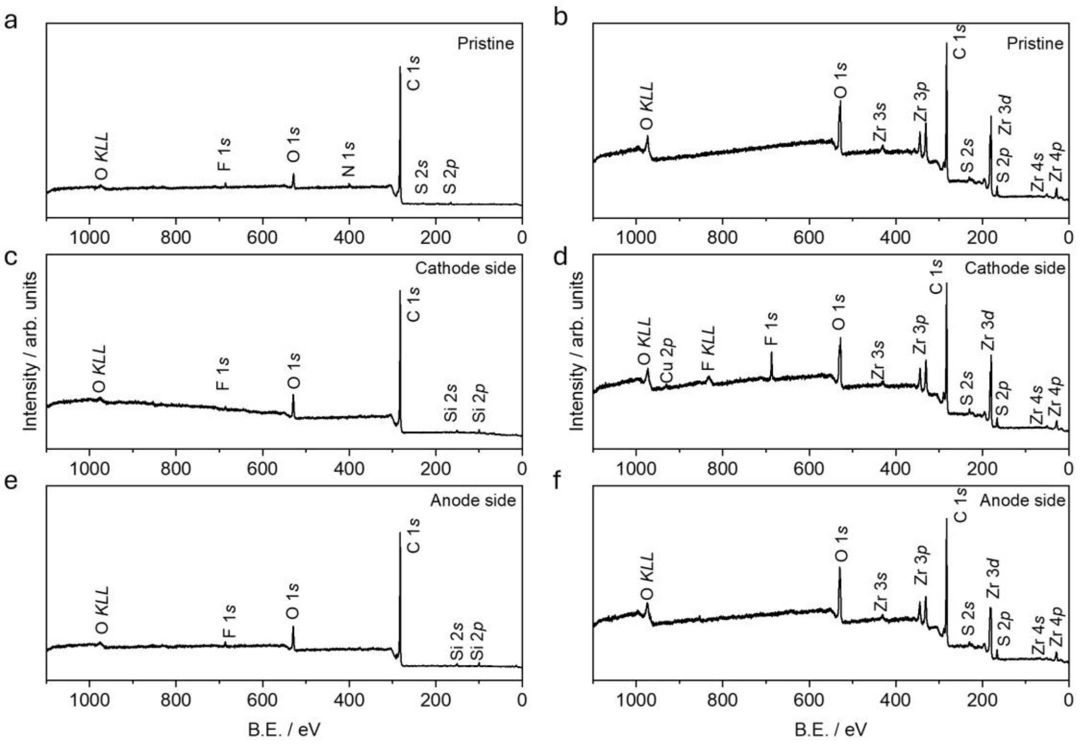

Supplementary Fig. 26 | a,b, Spectra of PiperION (a) and Zirfon 500+ (b) before testing. c,d, Spectra of the cathode-facing sides after testing for PiperION (c) and Zirfon 500+ (d). e,f, Spectra of the anode-facing sides after testing for PiperION (e) and Zirfon 500+ (f).

XPS experimental sample pretreat: After completion of the electrolysis, the AEM was carefully removed from the electrochemical cell and immediately rinsed with a large volume of Milli-Q water to remove any residual electrolyte from the membrane surface. Following the rinsing step, the AEM was dried in a vacuum desiccator until no visible moisture remained. The dried membrane was then subjected to further characterization.

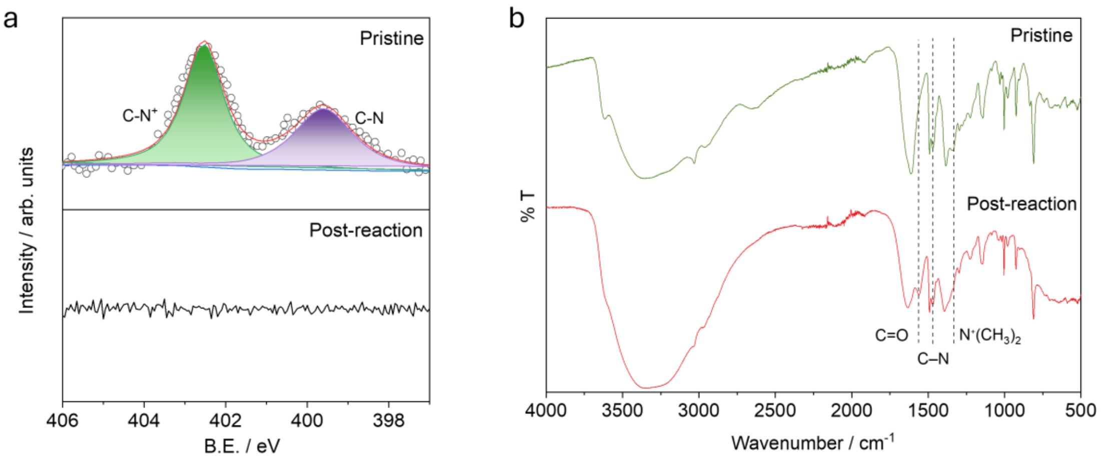

Supplementary Fig. 27 | N 1s fine scan and FTIR comparison of pristine PiperION and post-reaction PiperION.

To further elucidate the changes occurring in PiperION during CO electrolysis, we conducted a detailed analysis of the membrane before and after the stability test (200 mA cm^{-2}, 120 h) using high-resolution XPS and Fourier-transform infrared spectroscopy (FTIR). XPS surface analysis revealed a significant decrease in N content on the surface of PiperION. Notably, after the electrolysis, N could no longer be detected on the membrane surface, indicating substantial degradation or loss of nitrogen-containing functional groups.

The FTIR spectrum of the PiperION membrane after CO electrolysis. It revealed three key changes: (1) a significant decrease in the absorption intensity around 1450–1500 cm^{-1}. It corresponds to the bending vibrations of C–N and the wagging of N–CH_3 groups in quaternary ammonium structures, such as those found in piperidinium-based ionomers.[4] The diminished intensity in this region indicates potential degradation or de-functionalization of the quaternary ammonium groups, a phenomenon previously observed in polymeric materials bearing similar cationic moieties; (2) the disappearance of a distinct peak near ~1330 cm^{-1}. The loss of the ~1330 cm^{-1} peak—attributed to the symmetric bending vibration of methyl groups in $N^+(CH_3)_2$—provides further evidence of quaternary ammonium site decomposition.[5] and (3) the emergence of a new peak near ~1700 cm^{-1}. The enhanced peak near ~1700 cm^{-1} is characteristic of carbonyl (C=O) stretching vibrations, suggesting oxidative degradation or the formation of aldehyde/ketone groups. This carbonyl band is widely recognized as a marker of oxidative deterioration in polymers.[6] These functional group changes may result from interactions between organic electrolysis intermediates (e.g., carboxylic acids, alcohols, aldehydes) and the membrane polymer, further supporting the hypothesis that PiperION membranes are susceptible to structural degradation under CO electrolysis conditions.

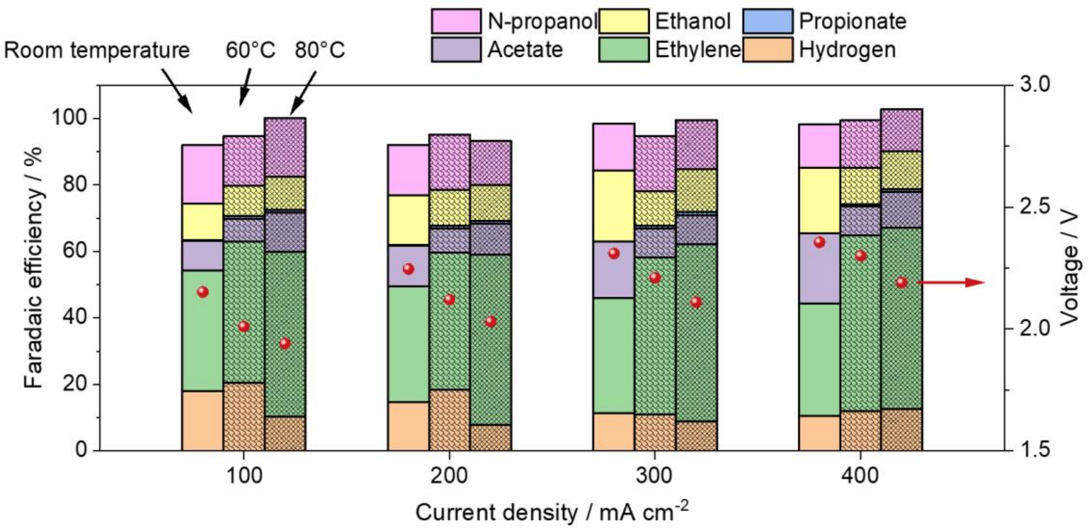

Supplementary Fig. 28 | Temperature-dependent tests of PiperION among different current density. The cell was operated among different current densities at different temperature, a 40–60 nm Cu nanoparticle cathode, a $NiFeO_x$/Ni foam anode, with CO fed at a rate of 50 sccm.

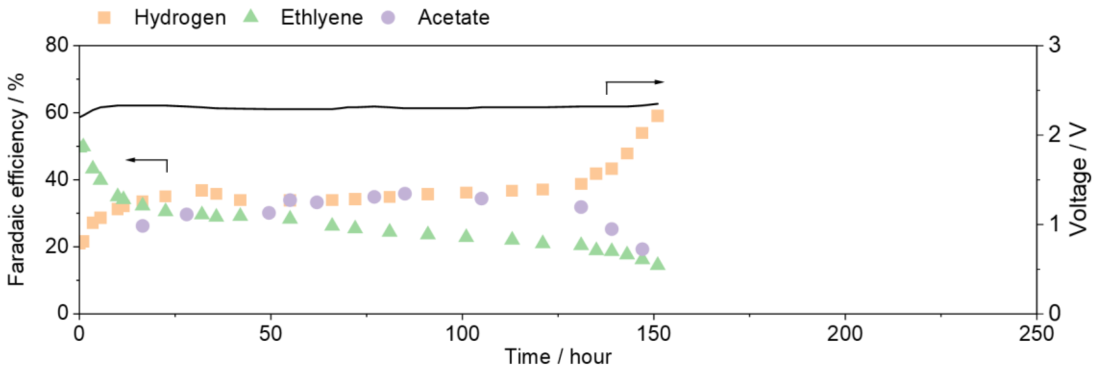

Supplementary Fig. 29 | Faradaic efficiency of acetate, ethylene and hydrogen and corresponding cell voltages measured using PiperION at a fixed current density of 200 mA cm^{-2}. The 5 cm^2 zero-gap CO electrolyzer was operated at 60°C with a 1 M KOH electrolyte at 3 mL min^{-1}, a 40–60 nm Cu nanoparticle cathode, a $NiFeO_x$/Ni foam anode, with CO fed at a rate of 50 sccm.

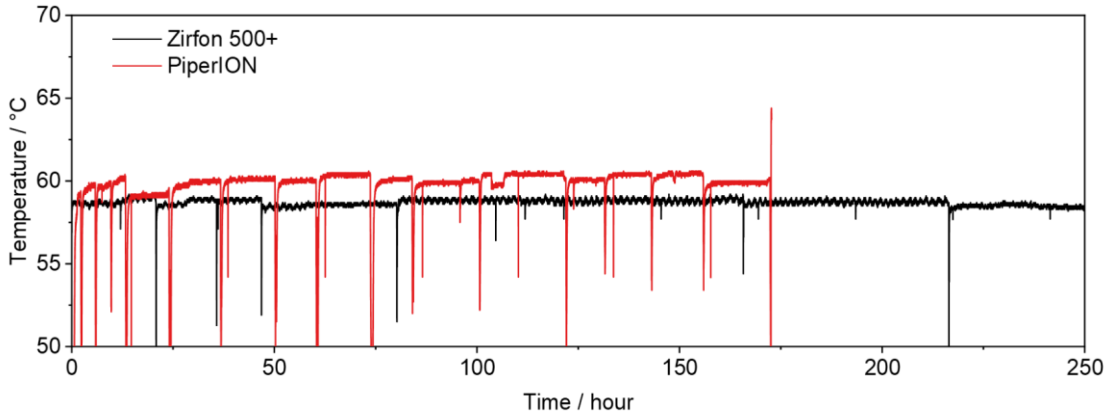

Supplementary Fig. 30 | Temperature profile of 5 cm^2 Zirfon 500+ based and PiperION-based cell of stability test at 60 °C. The sudden drop observed in the temperature profile was caused by the opening of the oven door during the sampling process.

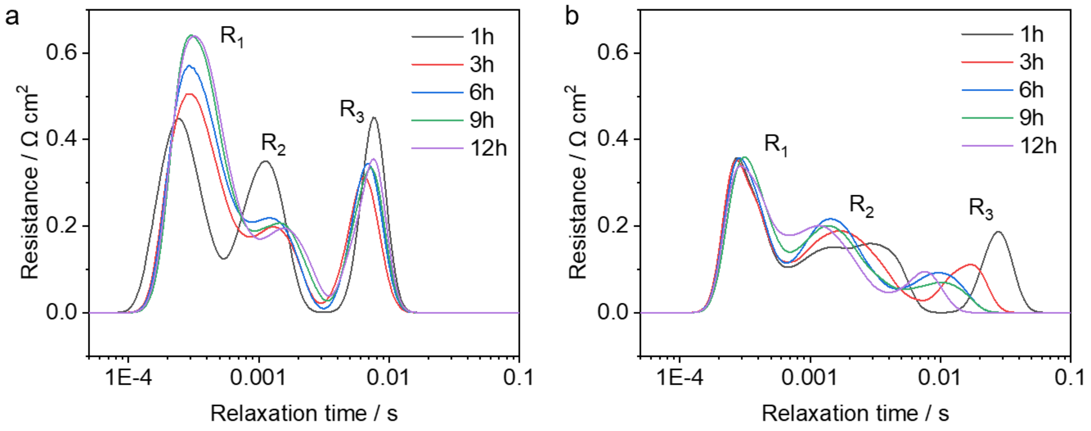

Supplementary Fig. 31 | DRT analysis of different resistances for (a) PiperION based cell and (b) Zirfon 500+ based cell among different reaction time at 60°C. R_1 is associated with electron and ionic resistance. R_2 is associated with HER, and R_3 with COER&OER.[7,8]

During the stability test at 60 °C, the R_1 resistance of the PiperION-based electrolyzer was found to gradually increase over time, whereas the R_1 of the Zirfon 500+ based cell remained essentially constant. Moreover, throughout the entire testing period, the R_1 of the PiperION based cell was consistently higher than that of the Zirfon-based system, despite the slightly superior intrinsic conductivity of the PiperION membrane. These observations suggest that the Zirfon 500+ diaphragm facilitates a more stable and effective cathode–diaphragm/membrane interface, thereby contributing to the superior COR performance observed in the Zirfon based cell.

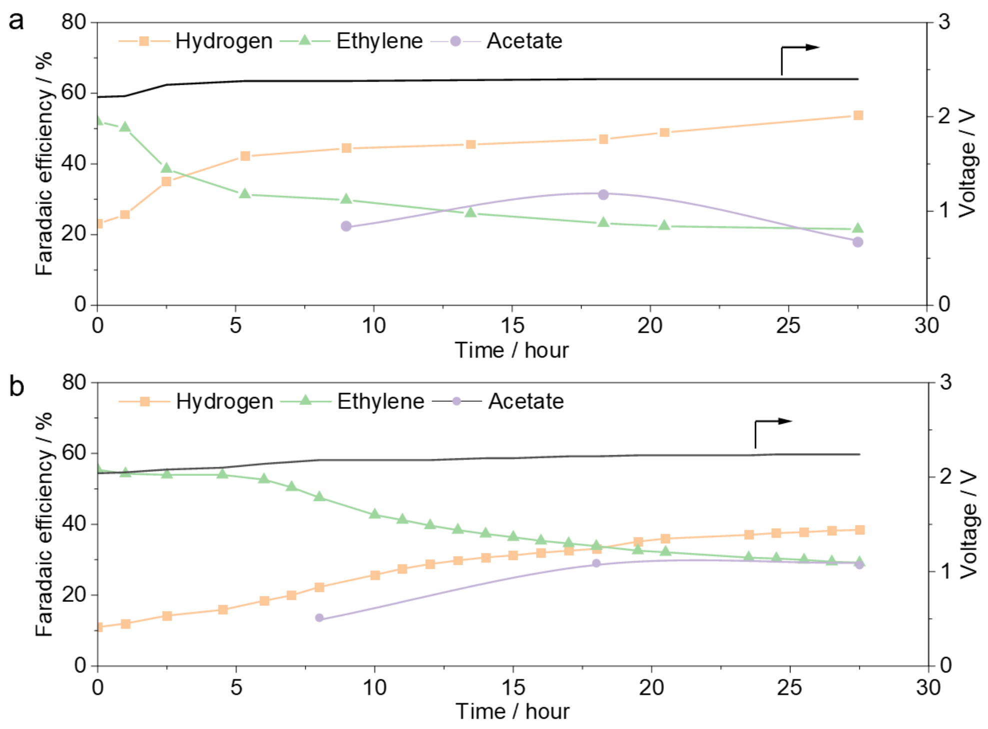

Supplementary Fig. 32 | Faradaic efficiency of acetate, ethylene and hydrogen and corresponding cell voltages measured using (a) PiperION and (b) Zirfon 500+ at a fixed current density of 200 mA cm^{-2}. The 5 cm^2 zero-gap CO electrolyzer was operated at 80°C with a 1 M KOH electrolyte at 3 mL min^{-1}, a 40–60 nm Cu nanoparticle cathode, a NiFeOx/Ni foam anode, with CO fed at a rate of 50 sccm.

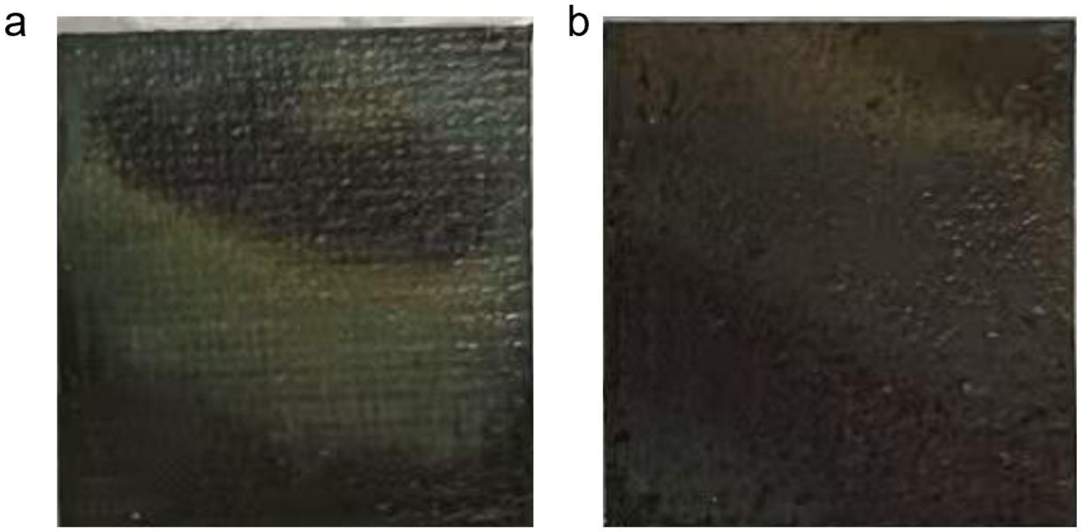

Supplementary Fig. 33 | Photographs of Cathode gas diffusion electrodes from (a) PiperION based cell and (b)Zirfon based cell after stability test at 80°C.

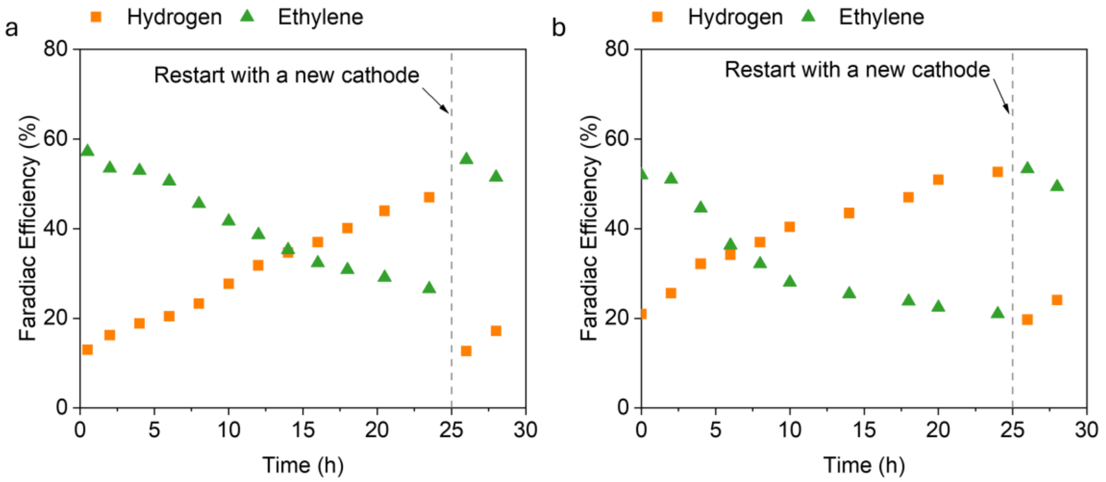

Supplementary Fig. 34 | Faradaic efficiency of ethylene and hydrogen measured using (a) Zirfon 500+ and (b) PiperION at a fixed current density of 200 mA cm^{-2}. Restart the cell with a new cathode at 25 h. To further elucidate the cause of cell failure at 80 °C, we conducted a 24-hour electrolysis experiment in 1 M KOH at a current density of 200 mA cm^{-2}. In both systems, the Faradaic efficiency for H_2 increased to approximately 50% after 24 hours of operation. However, upon replacing the cathode—while keeping the anode and diaphragm/AEM unchanged—the cell performance was fully restored to its initial state. This strongly indicates that cathode degradation is the primary cause of cell failure under elevated temperature conditions.

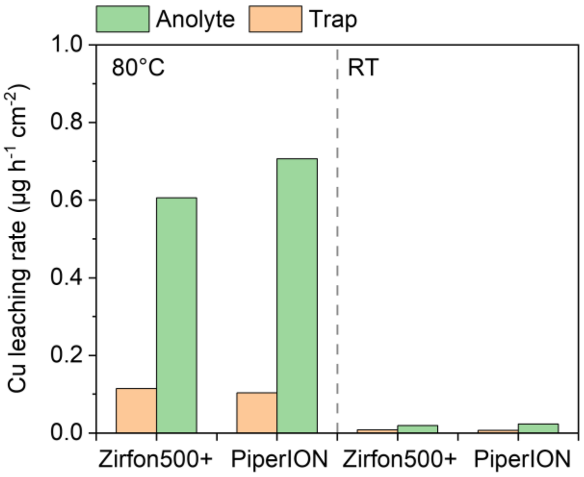

Supplementary Fig. 35 | Cu leaching rates at 80 °C and room temperature for both Zirfon based cell and PiperION based cell. Electrolyte samples from the anode side and the downstream trap solution on the cathode side after 24h test were collected to trace the fate of copper. We found that at 80 °C, both the Zirfon-based cell (with Cu leaching rates of 0.114 $\mu g\, h^{-1}\, cm^{-2}$ towards cathode and 0.606 $\mu g\, h^{-1}\, cm^{-2}$ towards anode) and the PiperION-based cell (0.103 $\mu g\, h^{-1}\, cm^{-2}$ towards cathode and 0.707 $\mu g\, h^{-1}\, cm^{-2}$ towards anode) exhibited significantly higher Cu dissolution rates compared to operation at room temperature. This indicates that elevated temperature accelerates the transformation of Cu into soluble species, such as $[Cu(OH)_4]^{2-}$, which likely contributes to its rapid deactivation.

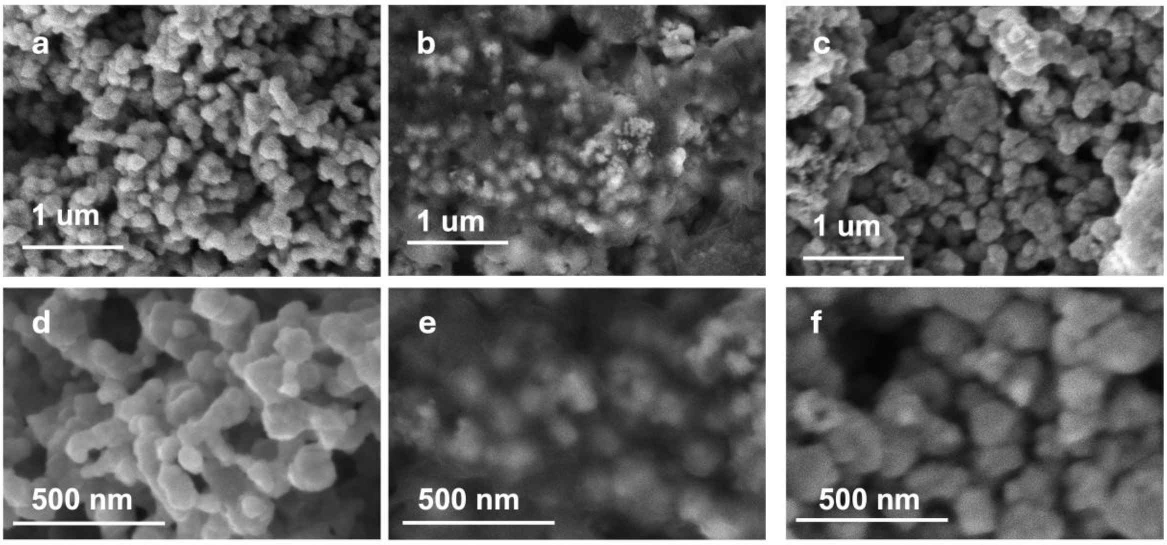

Supplementary Fig. 36 | Cathode before reaction (a and d), cathode after COR in 80°C for PiperION based cell (b and e) and Zirfon 500+ system (c and f), respectively. SEM imaging revealed substantial morphological changes in Cu particles on the cathode surfaces of both the Zirfon- and PiperION-based cells, indicating severe surface reconstruction. These observations suggest that cathode catalyst degradation is a major factor contributing to electrode failure under high-temperature conditions. Therefore, cathode degradation is likely the primary cause of cell failure in both systems operated at 80°C.

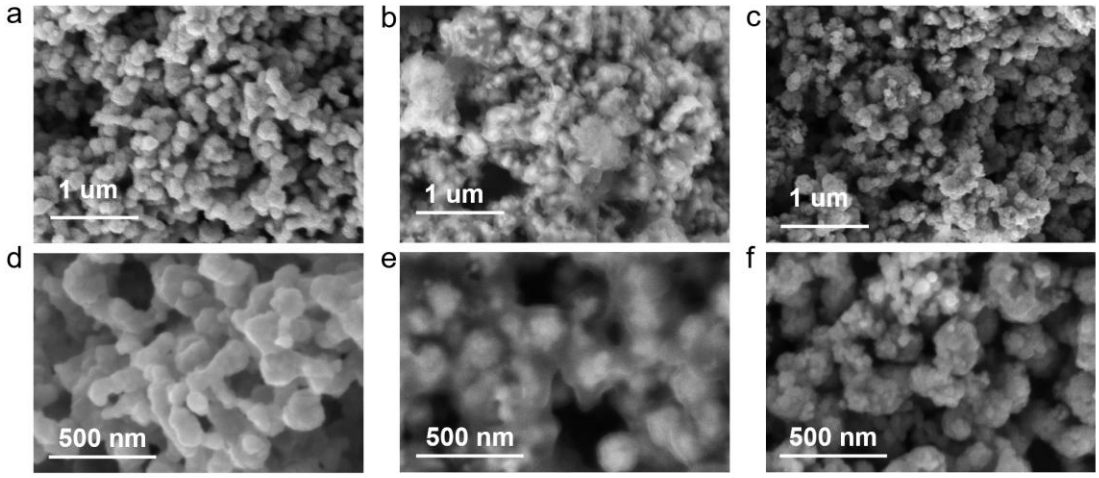

Supplementary Fig. S37 | Cathode before reaction (a and d), cathode after COR in 60°C for PiperION based cell (b and e, 140h) and Zirfon 500+ system (c and f, 300h), respectively.

After stability testing, the cathode surface of the PiperION based cell showed notable structural changes, whereas it is not obvious in the Zirfon case. The cathode in the Zirfon 500+ based cell maintained its nanoparticle morphology after long-term operation, indicating structural stability that may contribute to sustained electrochemical performance.

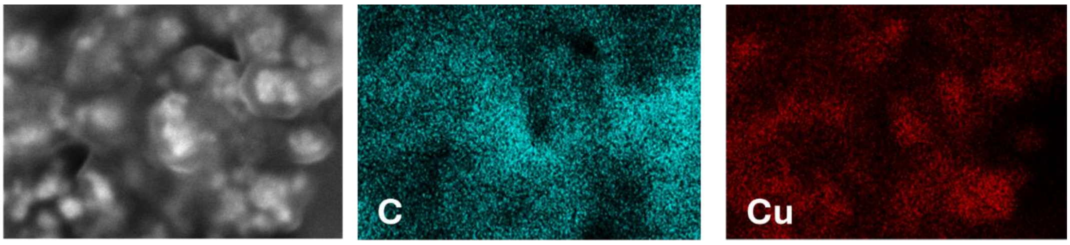

Supplementary Fig. 38 | Energy dispersive X-ray microanalysis (EDX) analysis of Cathode GDE after 60°C stability experiment in PiperION based cell.

To further investigate the changes occurring on the cathode surface of the PiperION based cell after the stability test, EDX analysis was performed. The results revealed the presence of carbonaceous species covering the Cu catalyst surface, which may account for the observed decline in product selectivity. Combined with XPS analysis of post-reaction cathodes, a significant increase in the C 1s peak was observed on the cathode surface of the PiperION based cell after stability test, indicating an accumulation of carbonaceous species. In addition, the Cu 2*p* peak intensity was markedly lower compared to that of the Zirfon 500+ based cell after reaction, further supporting the conclusion that the Cu catalyst surface was partially covered by carbon deposits. No Ni or Fe deposition was detected on the cathode surface after electrolysis, suggesting minimal anodic metal migration.

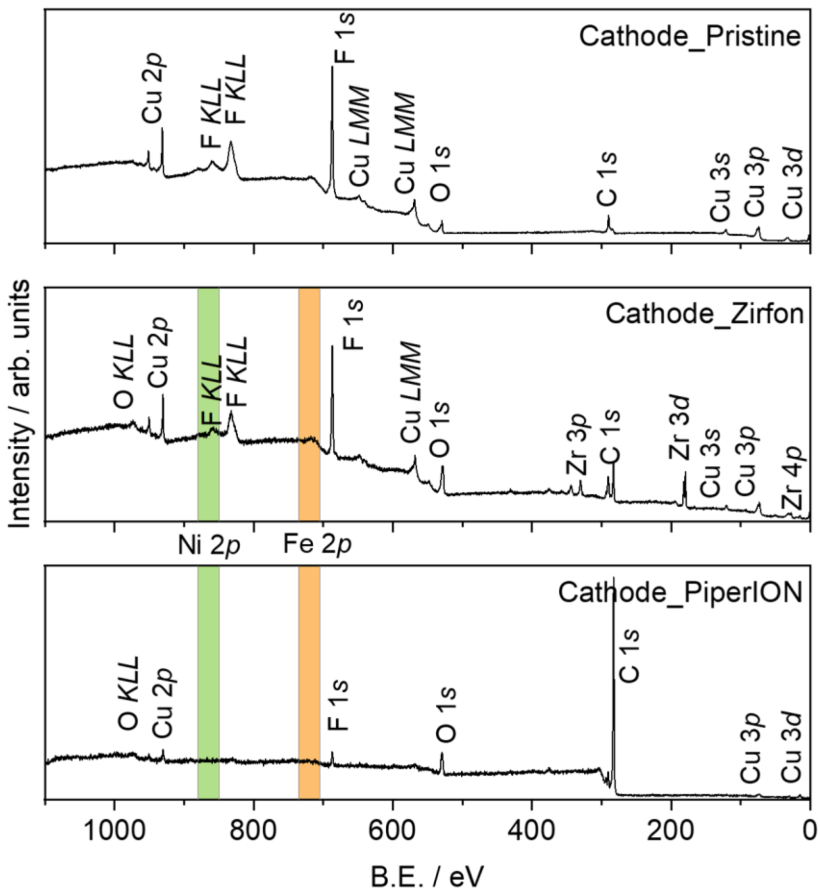

Supplementary Fig. 39 | XPS survey spectra of the cathode before and after (Zirfon 500+ and PiperION, respectively) 60°C stability test

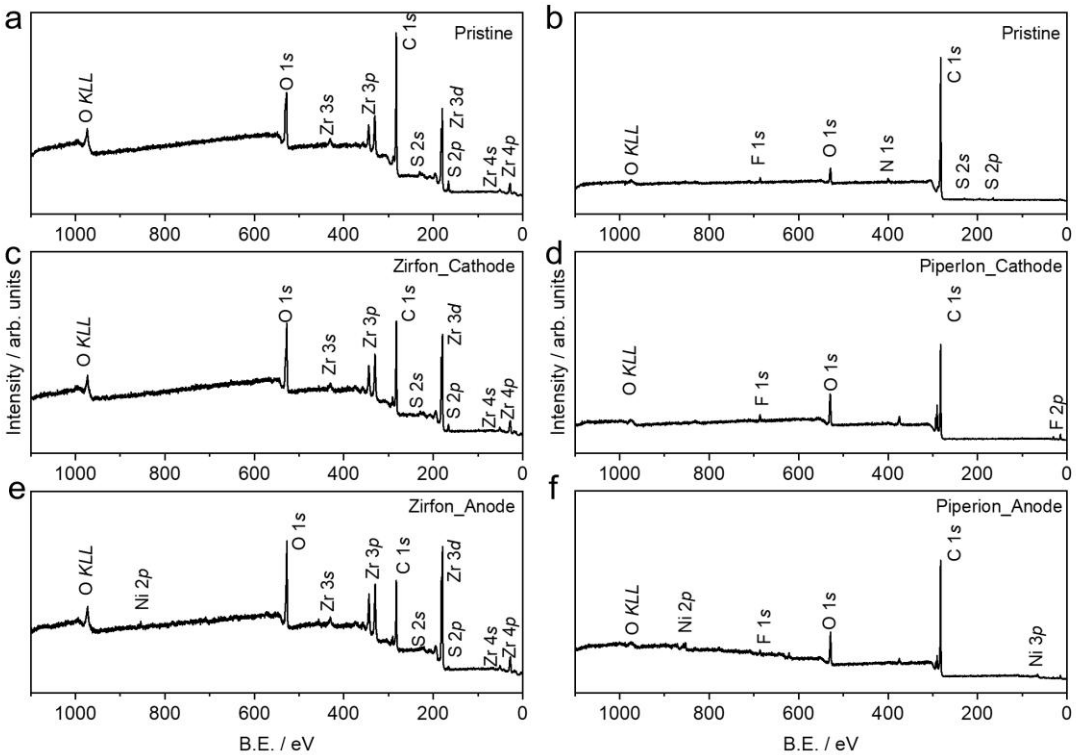

Supplementary Fig. 40 | a,b, XPS survey spectra of Zirfon 500+ (a) and PiperION (b) before testing. c,d, XPS survey spectra of the cathode-facing sides after testing for Zirfon 500+ (c) and PiperION (d). e,f, Spectra of the anode-facing sides after testing for Zirfon 500+ (e) and PiperION (f). The stability test was conducted under 60°C.

Post-reaction analyses of PiperION and Zirfon 500+ indicated that while macroscopic structural degradation was negligible, the N 1s peak in PiperION disappeared, aligning with degradation trends observed at room temperature. Additionally, Zirfon 500+ experienced significant zirconium content depletion in both surface and bulk regions, especially on the cathode-facing side. Specifically, the zirconium content decreased from 85 wt% to 36.5 wt% on the cathode-side surface and from 85 wt% to 69.3 wt% in the bulk (Supplementary Table S2). As known in AWE systems, reductions in ZrO_2 content can decrease water absorption capacity in Zirfon diaphragms,[9] potentially contributing to performance degradation and eventual failure during extended CO electrolysis operation.

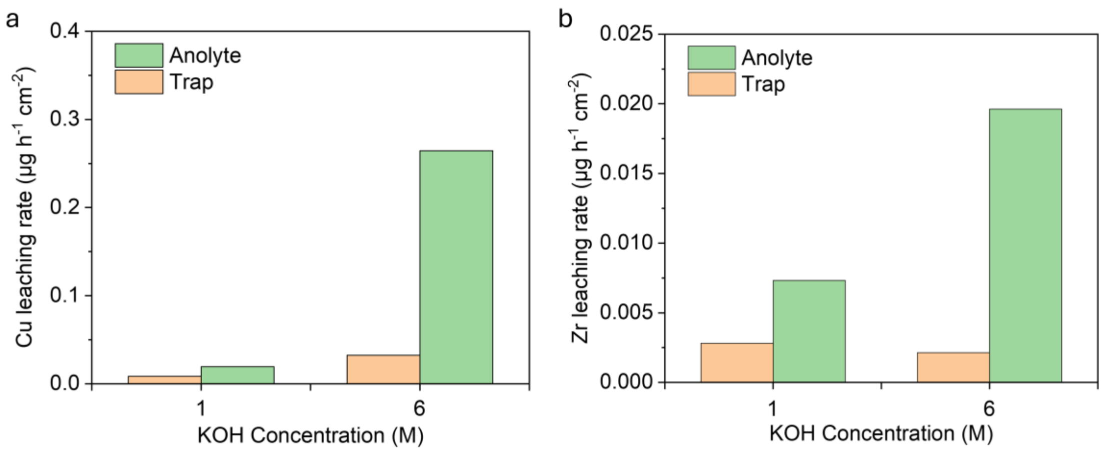

Supplementary Fig. 41 | Cu (a) and Zr (b) leaching rates under 1 M and 6 M KOH condition for both Zirfon based cell and PiperION based cell. We performed control experiments in both 1 M and 6 M KOH using Zirfon diaphragms and conducted 24-hour electrolysis at 200 mA cm^{-2}. ICP-MS analysis of electrolyte samples from the cathode effluent and anode compartments revealed only a modest increase in Zr dissolution in 6 M KOH compared to 1 M KOH (0.003 → 0.002 μg h^{-1} cm^{-2} at the cathode; 0.007 → 0.020 μg h^{-1} cm^{-2} at the anode). Importantly, the absolute Zr leaching rates remained very low, especially in comparison to Cu dissolution, which increased by nearly an order of magnitude under the same conditions. Furthermore, given that Zirfon 500+ contains ~42.5 mg_{Zr} cm^{-2}, this small degree of leaching is unlikely to impact diaphragm stability within the tested timeframes under 6 M anolyte condition. Besides, we note that Zirfon diaphragms have been widely used in commercial alkaline water electrolyzers operating in 6 M KOH for over 10000 hours without significant stability issues, suggesting that ZrO_2 dissolution is not a major concern under such conditions. We therefore conclude that while increased KOH concentration does slightly accelerate ZrO_2 dissolution, the primary degradation concern under high alkalinity lies with Cu instability rather than Zirfon degradation.

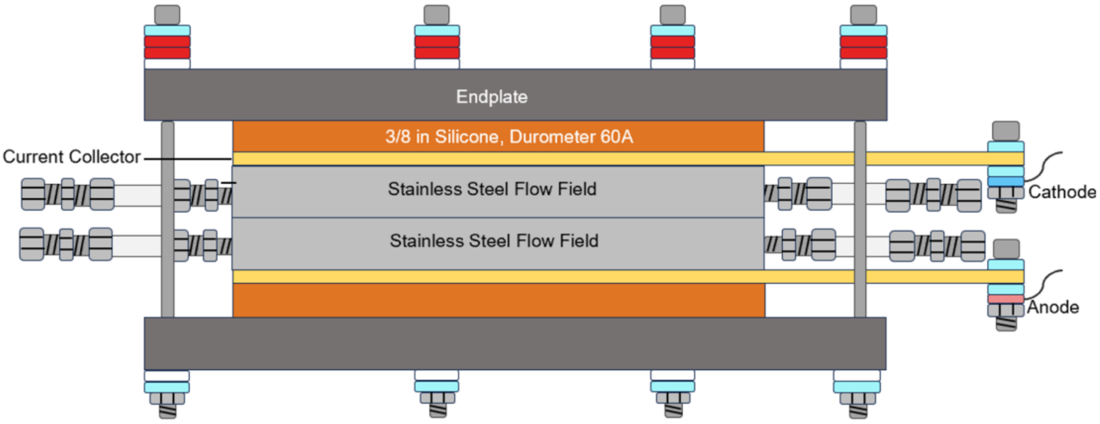

Supplementary Fig. 42 | Schematic diagram of the 100 cm^2 electrolyzer assembly components.

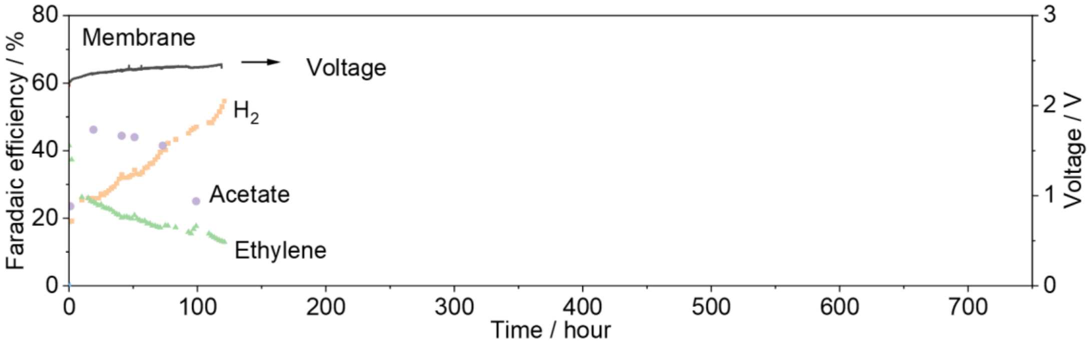

Supplementary Fig. 43 | Faradaic efficiency of acetate, ethylene and hydrogen and corresponding cell voltages measured using PiperION membrane at a fixed current density of 200 mA cm^{-2}. The 100 cm^2 zero-gap CO electrolyzer was operated with a 1 M KOH electrolyte at 60 mL min^{-1}, a 40–60 nm Cu nanoparticle cathode, a $NiFeO_x$/Ni foam anode, with CO fed at a rate of 400 sccm and at room temperature.

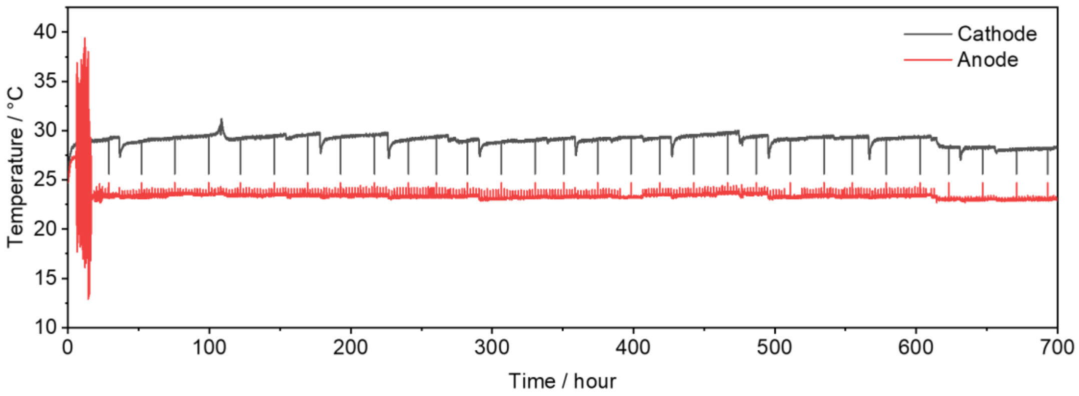

Supplementary Fig. 44 | Temperature profile of 100 cm^2 electrolyzer during 700-hour stability test. Initial signal fluctuations resulted from imperfect contact between the thermometer and the electrode.

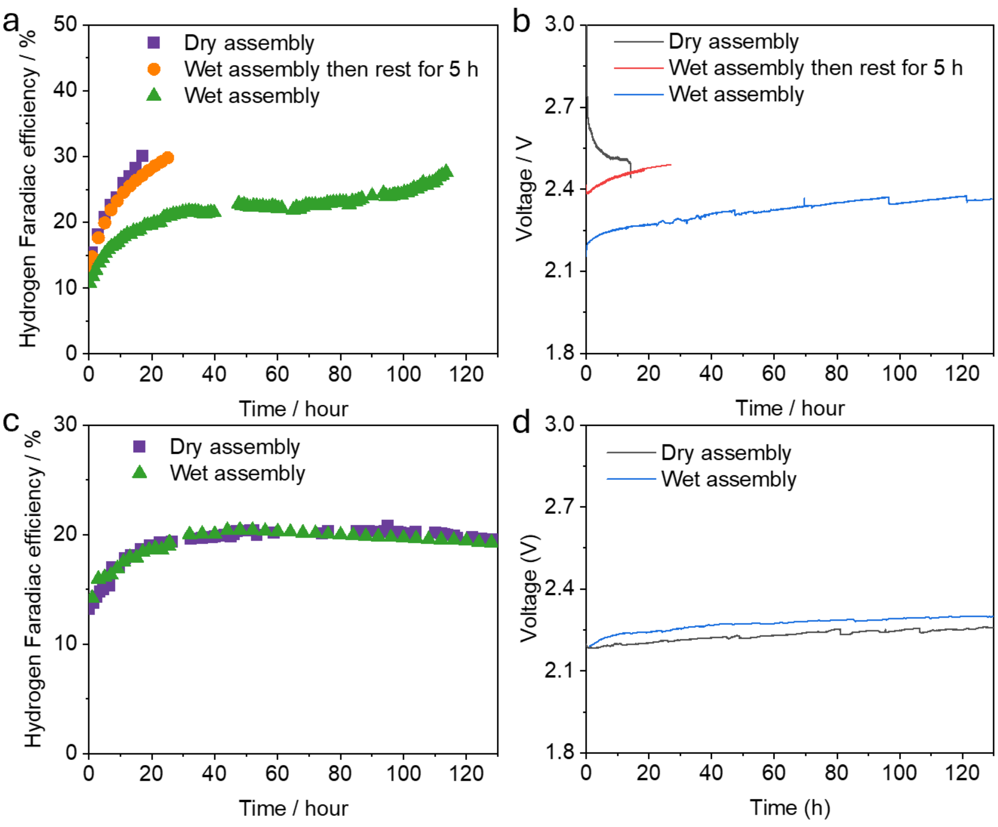

Supplementary Fig. 45 | Hydrogen Faradaic efficiency and cell voltage profiles of CO electrolyzers assembled using different methods. (a–b) Performance of PiperION based cell operated with three assembly conditions: dry assembly, wet assembly followed by 5 h resting before operation, and direct wet assembly. (c–d) Performance of Zirfon 500+ based cell assembled via dry and wet methods. These tests were designed to simulate practical constraints in industrial CO electrolyzer assembly.

In the dry assembly procedure, the activated AEM was air-dried until its dimensions and weight remained constant, then immediately integrated into the CO electrolyzer. PiperION based cells assembled in this way exhibited a rapid rise in H_2 Faradaic efficiency, reaching 30% within ~15 h, whereas the same level was only reached after ~120 h under standard wet-assembled conditions. Moreover, cells initiated with dry-assembled membranes displayed significantly higher initial voltages, which gradually decreased to ~2.6 V but remained elevated relative to the wet-assembled systems.

To further investigate the sensitivity of AEMs to pre-conditioning, a third protocol was tested in which the cell was assembled while the AEM remained hydrated, but left idle without electrolyte or CO gas for 5 h before initiating electrolysis. Even under these conditions, PiperION showed irreversible performance degradation, with H_2 FE reaching 30% after just 24 h. In contrast, Zirfon 500+ displayed negligible performance variation across all assembly conditions, indicating that its structure is not sensitive to handling time or hydration state. This robustness highlights its suitability for industrial stack assembly, where longer preparation times may be unavoidable.

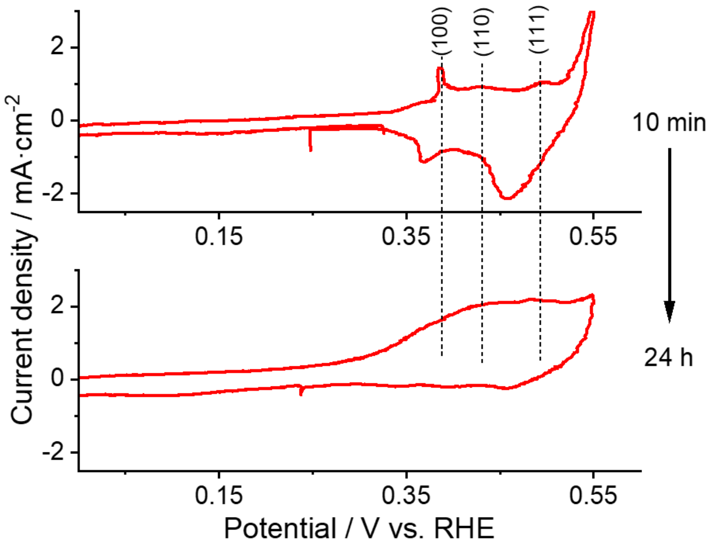

Supplementary Fig. 46 | CV test of the Cu catalyst for CO electroreduction reaction for 10 minutes and 24h. All Cu facet-related features, including (100), (110), and (111), were disappeared, indicating that the Cu catalyst is not stable under CO electroreduction reaction. The CVs were measured under Ar conditions at a scan rate of 20 mV s^{-1}. The resistance of the zero-gap electrolyzer was relatively low; therefore, the voltammograms reported here are presented without iR correction.

The experiment was conducted in a 1 M KOH with a 2.7 mL min^{-1} anolyte flow using a zero-gap electrolyzer as described previously. These experiments were performed using a BioLogic SP-300 potentiostat in a three-electrode configuration, with a polyethersulfone (PES) separator replacing a conventional membrane. A Hg/HgO reference electrode (Koslow Scientific, 5088 series, standard 1 molar solution) was inserted in the anolyte as part of the three-electrode configuration.

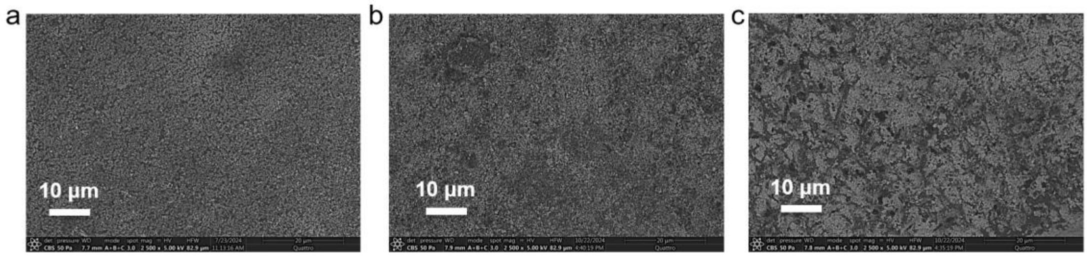

Supplementary Fig. 47 | Pristine Zirfon 500+ (a) and post-reaction Zirfon towards cathode side (b) and anode side (c). Post-reaction Zirfon was obtained from 100 cm^2 reactor after 700 hours stability test.

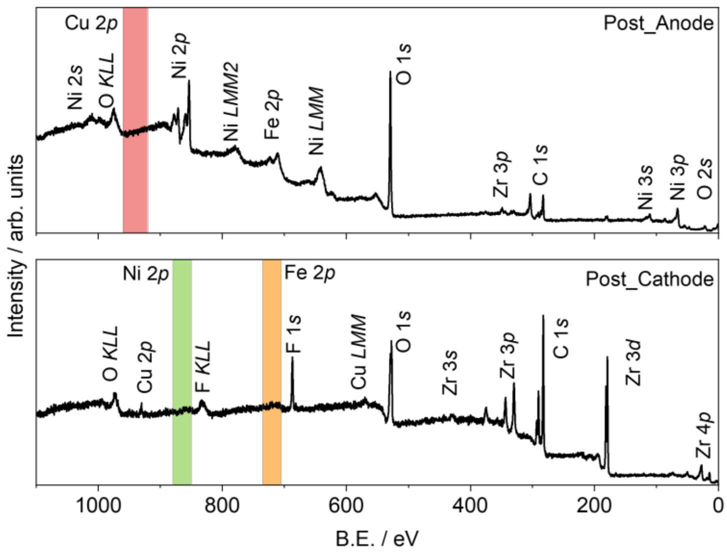

Supplementary Fig. 48 | XPS survey spectra of the cathode and anode after the stability test of the 100 cm² Zirfon 500+ reactor.

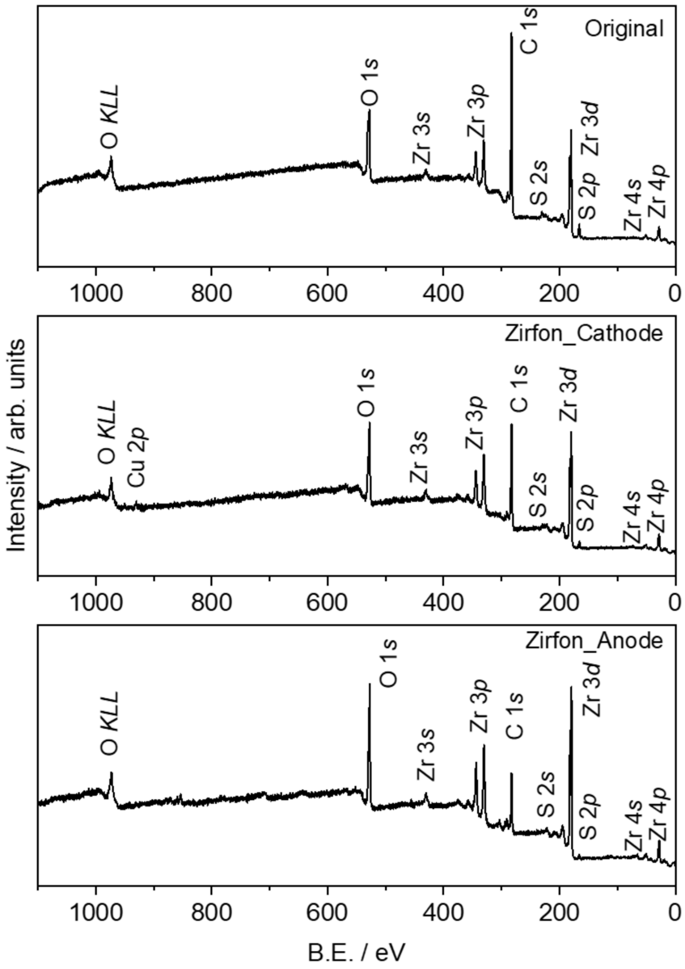

Supplementary Fig. 49 | Spectra of Zirfon 500+ before 700h stability testing, the cathode-facing sides and the anode-facing sides after 700h stability testing of the 100 cm² Zirfon 500+ reactor.

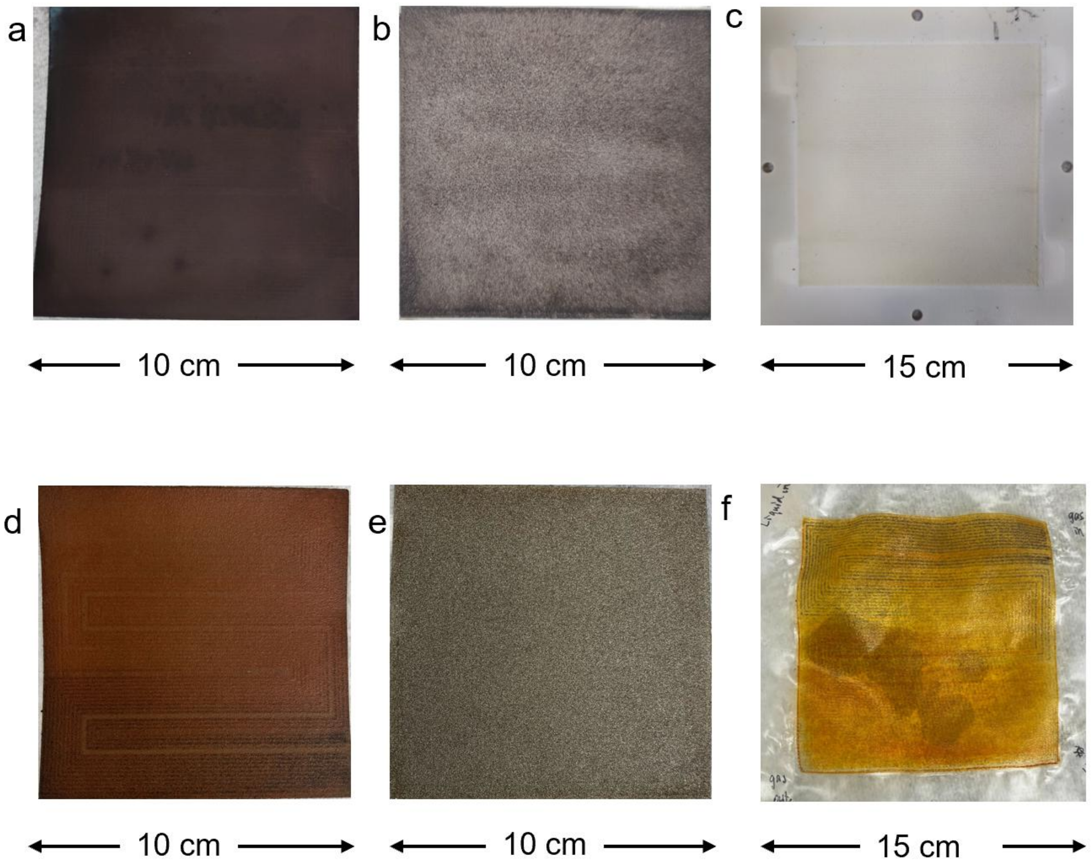

Supplementary Fig. 50 | Photograph of 100 cm^2 electrolyzer components of post-reaction from (a, b, and c) Zirfon 500+ and (d, e, and f) PiperION system.

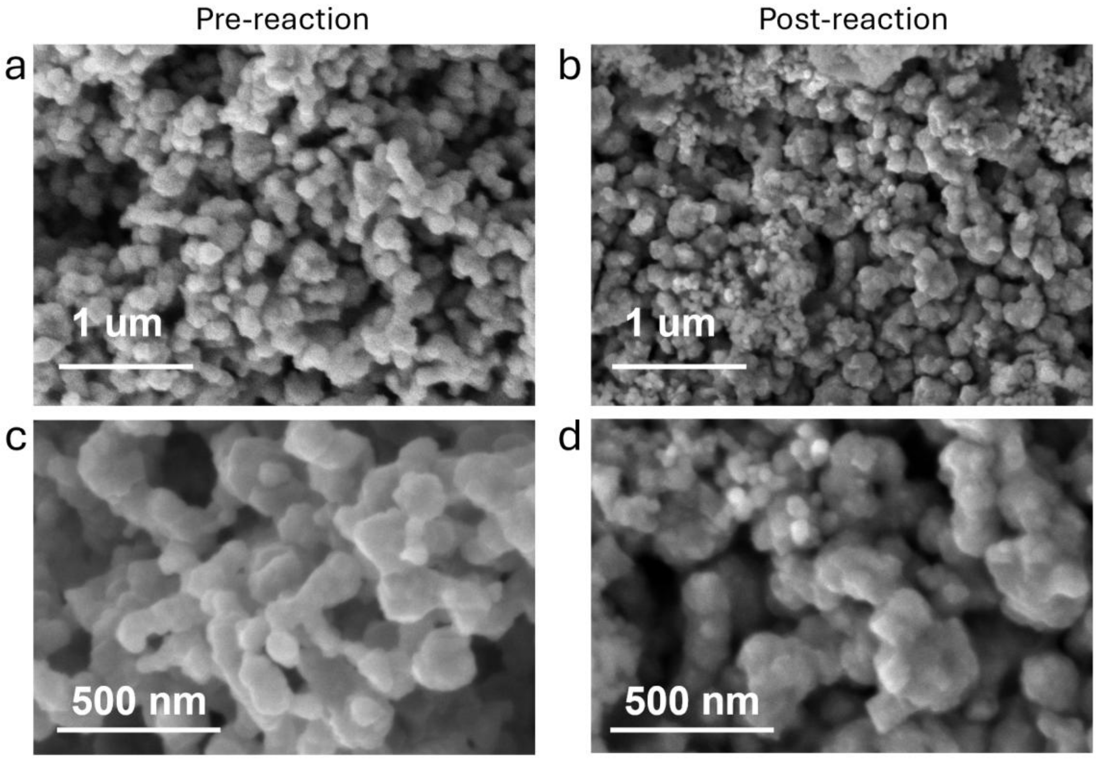

Supplementary Fig. 51 | SEM images of Cu cathodes before and after reaction in 100 cm2-COR electrolyzer assembled with Zirfon 500+ diaphragms for 700 hours at room temperature.

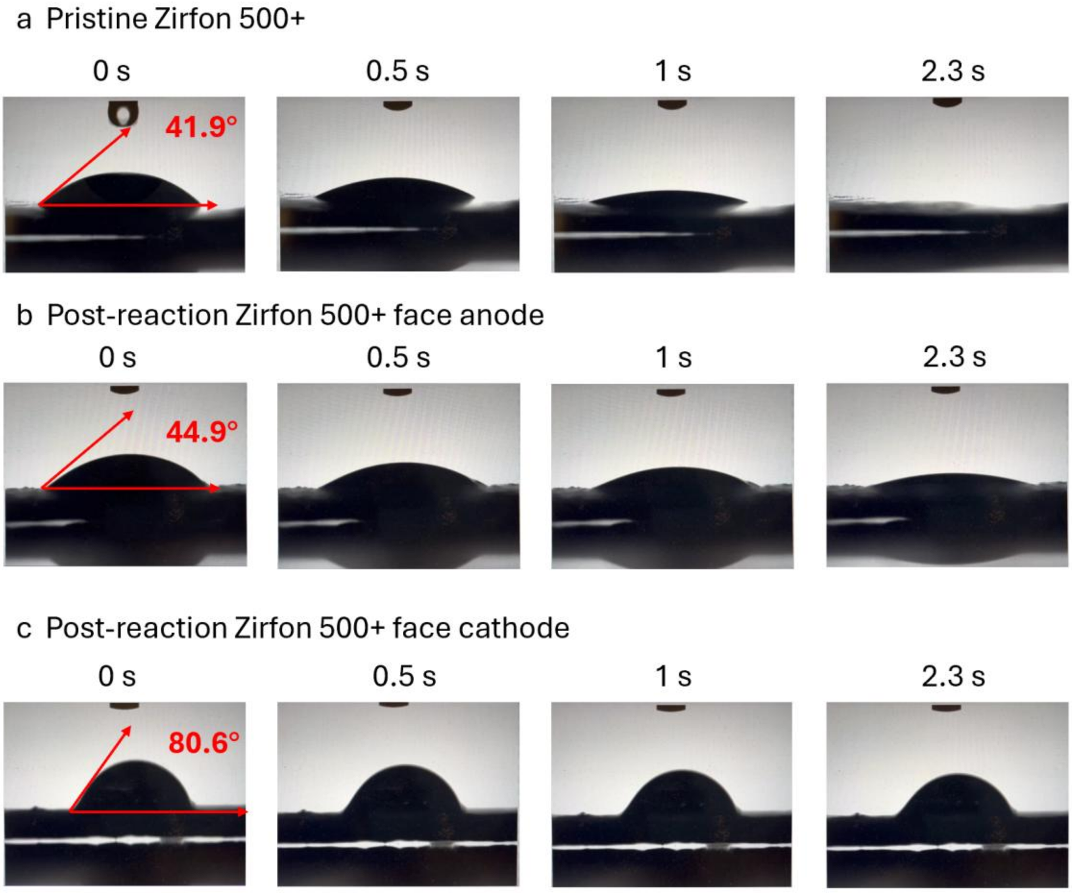

Supplementary Fig. 52 | Water contact angle measurements on the (a) pristine Zirfon 500+, as well as on the (b) anode-facing and (c) cathode-facing sides of the Zirfon 500+ retrieved from a 100 cm^2 electrolyzer.

Time-resolved images were captured from the initial moment of water droplet deposition (0 s) to 2.3 s. At 0 s, the pristine Zirfon 500+ exhibited a contact angle of 41.9°, indicating excellent hydrophilicity. After electrolysis, the anode-facing side showed a slightly increased contact angle of 44.9°, while the cathode-facing side exhibited a substantially higher value of 80.6°, suggesting significant loss of hydrophilicity. After 2.3 s, the water droplet on the pristine Zirfon 500+ was completely absorbed, confirming its highly hydrophilic nature. In contrast, the anode-facing side showed a contact angle approaching zero, but the droplet remained partially visible on the surface, indicating a slight reduction in water absorption capacity. Notably, the cathode-facing side retained a nearly unchanged droplet shape after 2.3 s, confirming a marked decrease in wettability.

The loss of hydrophilicity on the cathode side of the Zirfon 500+ diaphragm can critically impair the electrolyte–cathode interfacial contact. This deterioration is likely to increase interfacial overpotentials, hinder ion transport, and elevate the risk of gas crossover, thereby accelerating electrolyzer degradation during extended operation.

Supplementary Table S1 | Comparison of the physical properties of different Zirfon diaphragms

	Zirfon 220	Zirfon 500	Zirfon 500+
Thickness (μm)	220±30	500±50	500±50
Highest operation temperature (℃)	100	110	110
Porosity (%)	60±10	55±10	60±5
Bobble point (psi)	25.8	48.5	50
Tensile strength (Mpa)	20	25	30

Supplementary Table S2 | Zr percentage of post-reaction Zirfon at cathode and anode side after 60 °C stability test experiments

Zirfon sample	wt% from EDX	Atom% from EDX	wt% from XPS	Atom% from XPS
Pristine	85.0	42.7	83.5	40.32
Post-reaction sample face cathode side	69.3	22.6	36.5	7.48
Post-reaction sample face anode side	84.0	40.9	44.9	11

Supplementary Table S3 | Zr percentage of post-reaction Zirfon 500+ at cathode and anode side after 100 cm^2 electrolyzer stability test experiments

Zirfon sample	wt% from EDX	Atom% from EDX	wt% from XPS	Atom% from XPS
Pristine	85.0	42.7	83.5	40.32
Post-reaction sample face cathode side	65.3	19.8	50.3	11.78
Post-reaction sample face anode side	80.2	34.8	70.5	23.97

To further elucidate the mechanism underlying Zr loss during in situ CO electrolysis testing, we conducted a 24-hour electrolysis experiment in 1 M KOH electrolyte at a current density of 200 mA cm^{-2}. Electrolyte samples from the anode side and the downstream trap solution on the cathode side were collected to trace the migration pathway of lost Zr. ICP-MS analysis revealed detectable Zr dissolution even in the early stages of electrolysis, with a total Zr loss rate of approximately 0.01 μg h^{-1} cm^{-2}. Specifically, about 0.0075 μg h^{-1} cm^{-2} of Zr was found to migrate into the anolyte, while approximately 0.0026 μg h^{-1} cm^{-2} was captured in the cathodic trap. Based on the post-test XPS analysis, which shows that the Zr content on the cathode-facing side of the Zirfon 500+ (65.3 Wt%) is significantly lower than that on the anode-facing side (80.2 Wt%), it can be inferred that most of the dissolved Zr on the cathode side diffuses across the diaphragm and accumulates in the anolyte, while only a small portion is carried out by the gas stream and collected in the cathodic trap. These results provide direct evidence that ZrO_2 leaches progressively from the Zirfon 500+ during electrochemical operation, ultimately leading to the degradation of its hydrophilic properties over time.

Supplementary Table S4 | Comparison of Zirfon 500+ properties before and after long-term CO electrolysis

	Cathode-side wettability (degree)	Tensile strength (MPa)	Resistance (Ω cm^2)	Bubble point (psi)
Pristine sample	41.9	25.7	0.59	50
Post reaction sample	80.6	23.8	0.67	16.2

To further evaluate the impact of Zirfon 500+ diaphragm aging on its overall performance, we compared key properties of pristine and aged samples after 700 hours of stability testing in a 100 cm^2 CO electrolysis cell (Supplementary Table S4). The post-test diaphragm exhibited a slight decrease (~10%) in tensile strength compared to the fresh sample, indicating minor mechanical degradation while still retaining adequate structural integrity. Notably, the hydrophilicity of the cathode-facing side declined significantly, with the water contact angle increasing from 41.9° to 80.6°, which aligns with the observed reduction in ZrO_2 content on the cathode side. Additionally, the measured resistance of the diaphragm increased after long-term testing, suggesting that electrolyte transport through the diaphragm became more restricted. A pronounced decrease in bubble point was also observed, dropping from an initial 50 psi to 16.2 psi. This reduction is particularly critical, as the back pressure on the cathode side during testing was set to 17 psi, which correlates well with the experimentally observed gas crossover from cathode to anode during the final stage of electrolysis.

Supplementary Note 1 | Energy efficiency and CO utilization

1. Energy Efficiency (EE)

The Faradaic efficiency (FE) for each product was calculated using the equation:

$$\mathrm{FE} = \frac{n \cdot F \cdot C \cdot V}{Q} \times 100\% \quad (5)$$

where

- n is the number of electrons transferred per molecule of product,
- F is the Faraday constant (96,485 C mol^{-1}),
- C is the concentration of product (mol L^{-1}),
- V is the volume of electrolyte (L),
- Q is the total charge passed during electrolysis (C), calculated from the applied current and operation time.

For gaseous products, the amount (mol) was calculated from GC calibration and converted to charge contribution based on their electron number. All detectable products including H_2, C_2H_4, acetate, ethanol, n-propanol, and propionate were included in the total FE calculation.

The energy efficiency (EE) for each product was calculated using the following equation:

EE = (E° / E_cell) × FE (6)

where E° is the standard thermodynamic potential of the product, E_cell is the full cell voltage (2.2 V in our case), and FE is the Faradaic efficiency of the product.

For acetate and ethylene, the standard potential is approximately 0.08 V vs. RHE. Based on the long-term stability data, the average Faradaic efficiencies were ~45% for acetate and ~15% for ethylene.

Thus:

EE for acetate: (0.08 / 2.2) × 45% = 1.64% (7)

EE for ethylene: (0.08 / 2.2) × 15% = 0.55% (8)

- Total energy efficiency from carbon products: EE_total = 1.64% + 0.55% = 2.19%

2. CO Utilization Efficiency

The CO inlet flow rate was 400 sccm, equivalent to 0.4 L/min. Under standard conditions (22.4 L/mol), this corresponds to:

CO_in = 0.4 / 22.4 ≈ 0.01786 mol/min (9)

To estimate how much CO was consumed, we first calculated the total number of electrons transferred based on the assumed operating current density of 200 mA/cm^2 over a 100 cm^2 electrode area:

$$I = 200 \times 100 = 20 \text{ A} \quad (10)$$

$$n\_e = 20 / 96485 \approx 0.01244 \text{ mol } e^-/\text{min} \quad (11)$$

Using the number of electrons required per product molecule (8 e^- for acetate, 12 e^- for ethylene), and 45% FE for acetate and 15% FE for ethylene, the product formation rates are:

$$\text{Acetate: } (0.01244 \times 0.45) / 8 \approx 0.00070 \text{ mol/min} \quad (12)$$

$$\text{Ethylene: } (0.01244 \times 0.15) / 12 \approx 0.00016 \text{ mol/min} \quad (13)$$

Corresponding CO consumption (2 mol CO per molecule of acetate or ethylene):

$$\text{Acetate: } 2 \times 0.00070 = 0.0014 \text{ mol CO/min} \quad (14)$$

$$\text{Ethylene: } 2 \times 0.00016 = 0.00032 \text{ mol CO/min} \quad (15)$$

- Total CO consumed ≈ 0.0017 mol/min

Therefore:

$$\text{CO Utilization} = (0.0017 / 0.01786) \times 100\% \approx 9.5\% \quad (16)$$

Reference

1 Bohn, L. *et al.* Reference electrode types for zero-gap CO_2 electrolyzers: benefits and limitations. *Adv. Sci.* ***11***, 2402095 (2024).

2 Hansen, K. U., Cherniack, L. H. & Jiao, F. Voltage loss diagnosis in CO_2 electrolyzers using five-electrode technique. *ACS Energy Lett. 7*, 4504-4511 (2022).

3 Heinzmann, M., Weber, A. & Ivers-Tiffée, E. Advanced impedance study of polymer electrolyte membrane single cells by means of distribution of relaxation times. *Journal of Power Sources* ***402***, 24-33 (2018).

4 Prakalathan, K., Mohanty, S. & Nayak, S. K. Rheological and thermomechanical behaviour of polylactide/hexadecyl trimethyl ammonium–modified layered silicate nanocomposites. *J. Thermoplast. Compos. Mater.* ***27***, 1303-1320 (2014).

5 Rout, A., Kumar, S. & Ramanathan, N. Understanding the coordination behavior of quaternary ammonium and phosphonium nitrate ionic liquids under gamma irradiation: A combined spectroscopic investigation. *J. Mol. Liq.* ***367***, 120484 (2022).

6 Celina, M., Ottesen, D. K., Gillen, K. T. & Clough, R. L. FTIR emission spectroscopy applied to polymer degradation. *Polym. Degrad. Stab.* ***58***, 15-31 (1997).

7 Cherniack, L. H. *et al.* An interfacial engineering approach toward operation of a porous solid electrolyte CO_2 electrolyzer. *ACS Energy Lett.* ***10***, 1508-1516 (2025).

8 Qu, Y. *et al.* Operando diagnosis of MEA-type CO_2 electrolyzer via distribution of relaxation times analysis. *ACS Energy Lett.* ***9***, 3042-3048 (2024).

9 Vermeiren, P., Adriansens, W., Moreels, J. P. & Leysen, R. Evaluation of the Zirfon® separator for use in alkaline water electrolysis and Ni-H2 batteries. *International Journal of Hydrogen Energy* ***23***, 321-324 (1998).
